# Ecosystem, spatial and trophic dimensions of niche partitioning among freshwater fish predators

**DOI:** 10.1186/s40462-025-00559-0

**Published:** 2025-05-27

**Authors:** Milan Říha, Lukáš Vejřík, Rubén Rabaneda-Bueno, Ivan Jarić, Marie Prchalová, Ivana Vejříková, Marek Šmejkal, Petr Blabolil, Martin Čech, Vladislav Draštík, Michaela Holubová, Tomáš Jůza, Karl Ø. Gjelland, Zuzana Sajdlová, Luboš Kočvara, Michal Tušer, Jiří Peterka

**Affiliations:** 1 Biology Centre of the Czech Academy of Sciences, Institute of Hydrobiology, České Budějovice, Czech Republic; 2 Université Paris-Saclay, CNRS, AgroParisTech, Ecologie Systématique Evolution, Gif- sur-Yvette, France; 3 Faculty of Science, University of South Bohemia in České Budějovice, České Budějovice, Czech Republic; 4 Norwegian Institute for Nature Research (NINA), Tromsø, Norway

**Keywords:** Niche partitioning, Resource utilization, Interspecific competition, Spatial niches, Trophic niches, Environmental variation, Dietary plasticity, Predator interactions, Aquatic ecosystems, Acoustic telemetry, Stable isotopes

## Abstract

**Background:**

Niche partitioning allows species to diversify resource utilisation and space allocation and reduce interspecific competition. Variations in abiotic and biotic conditions in different ecosystems may further influence resource availability and habitat utilisation, potentially reducing competition. The aim of this study is to investigate the effects of environmental variation on spatial and trophic niche overlap between two freshwater apex predators, the northern pike (*Esox lucius)* and the European catfish (*Silurus glanis*), in three different water bodies.

**Methods:**

We used fine-scale acoustic telemetry to assess the spatial niche overlap of pike and catfish, analyzing their spatial and habitat use in relation to the thermocline and their presence in benthic versus open-water habitats. Stable isotope analysis (SIA) was used to quantify trophic niche overlap and dietary differences between the species. We compared the habitat use, spatial niche width and overlap, and trophic differentiation among waterbodies to determine how environmental conditions influence predator interactions.

**Results:**

During summer, pike and catfish primarily occupied benthic habitats above the thermocline across all waterbodies and diel periods. However, catfish more frequently used open water above the thermocline, while pike were more often present in both open water and benthic habitats below it. While this general pattern of habitat use was consistent, its extent varied among lakes, suggesting that local environmental conditions shape species-specific habitat selection. Despite these variations, the species exhibited substantial spatial overlap, though its magnitude fluctuated across waterbodies and diel periods. Catfish occupied a broader spatial niche in two waterbodies, while pike had a broader niche in one. Across all lakes, catfish consistently maintained a broader trophic niche than pike. However, pike exhibited higher trophic overlap with catfish than vice versa, with nearly complete overlap in one lake and substantial but incomplete overlap in others. This suggests that pike relies more heavily on shared prey resources, while catfish exploits a broader range of food sources beyond those used by pike.These patterns were primarily driven by the position of the thermocline, prey availability, structural complexity and the greater foraging plasticity of catfish, highlighting the environmental dependence of niche partitioning in these predators.

**Conclusions:**

Our findings demonstrate that spatial and trophic niche overlaps between pike and catfish are highly context-dependent, shaped by abiotic conditions, prey availability, and species-specific foraging strategies. This study highlights the importance of integrating spatial and trophic analyses to understand predator interactions in aquatic ecosystems.

**Supplementary Information:**

The online version contains supplementary material available at 10.1186/s40462-025-00559-0.

## Background

Niche partitioning is a fundamental ecological concept that explains how different species within a community divide and utilize available space and resources to reduce interspecific competition and coexist [1]. It has been demonstrated across a wide range of taxa [2–6] and it is an essential driver in shaping community structure and dynamics [7, 8]. Species can reduce competition through various mechanisms, including shifts in habitat, diet, or timing of activity [4, 9–12]. Such diversification of prey and resource use helps to avoid competitive exclusion and facilitate coexistence [12, 13].

In addition to spatial and trophic strategies, temporal niche partitioning—where species differ in the timing of their activities—has emerged as an important, yet underexplored, mechanism for reducing overlap in resource use [12, 14]. Temporal differences, such as diurnal versus nocturnal activity or even more nuanced crepuscular patterns, allow coexisting species to exploit similar habitats and food sources at different times [15]. The degree of temporal partitioning can be influenced by species-specific circadian rhythms [12]. Thus, the intensity of overall niche partitioning may depend not only on the degree of spatial or trophic overlap but also on species temporally segregation.

The intensity of niche partitioning depends on the degree of overlap between species, their competitive ability [16] or individual characteristics [17–19]. Recent research has also emphasized the importance of environmental conditions for niche partitioning [20]. Different abiotic and biotic conditions across ecosystems can affect the availability of resources and space for conspecifics, impacting their ability to shift habitat or diet to avoid competition. Consequently, niche overlap can be environmentally dependent, leading to local variations in competition levels and causing local adaptations. For example, trophic niche overlap in aquatic environments can be changed with different turbidity or turbulence levels [21, 22], or by differences in ecosystem structural complexity provided by macrophytes [23]. However, studies explicitly examining the context dependence of niche partitioning remain rare.

There is a need for studies across multiple ecosystems to better understand how environmental variability influences niche partitioning and, in turn, how this may affect ecosystem functioning. In addition, the integration of advanced technologies such as stable isotope analysis and in situ behavioural observation may help to elucidate the complex interactions between environmental conditions and multi-species behaviour [24, 25]. These approaches can improve predictions of conspecific interactions, detect resulting changes in community structure, and evolutionary mechanisms for local adaptations under different environmental context.

The European catfish (*Silurus glanis*) and the northern pike (*Esox lucius*) are among the most important freshwater predators in the Holarctic region (catfish and pike hereafter in the text) [26, 27]. Both predators often live in sympatry and largely share their diet and space [28–30]. The pike is a voracious hunter that feeds on a variety of fish and other prey, and its introduction can have a significant impact on fish species composition [27, 31]. Similarly, the catfish is primarily a piscivore [26], but numerous studies have highlighted its ability to adjust to different food sources and exhibit generalist foraging behaviour [32]. While pike is typically associated with structurally complex habitats and is often used as a classic example of a diurnal, sit-and-wait ambush predator, recent studies have shown that it is able to dynamically change its habitat use depending on the environment [33–35]. The catfish is an active nocturnal hunter that pursues its prey in both nearshore and offshore habitats [36, 37]. It exhibits a high flexibility in changing its foraging strategy [28, 38, 39] and tends to favor warmer water compared to pike [32]. Both species are important for the functioning and management of ecosystems, as well as important commercial and game species [27, 32]. The catfish is expanding into new freshwater ecosystems, where its presence can alter food web dynamics, restructure predator-prey interactions, and impact biodiversity by exerting strong predation pressure on native species, including fish, amphibians, and waterfowl [26, 40, 41].

Despite their importance and numerous studies on both species [26, 27, 32, 42], there is little information on their spatial partitioning in different environments. Our previous studies on these predatory fish have shown that their activity, space utilisation and temporal dynamics depend on the environment and vary greatly from lake to lake [29, 34, 43]. The same applies to foraging behaviour and the extent and position of trophic niches [28, 44]. Such behavioural changes were associated with differences in structural complexity and/or prey type and availability [28, 34, 43, 44]. Furthermore, our previous studies indicated potentially large overlaps in the spatial and trophic niche dimensions of these predators, although the intensity of these overlaps may depend on the lakes and their local conditions [28, 34, 43, 44]. In addition, recent evidence indicates that the extent of their niche overlap may also be influenced by their differential use of benthic and pelagic habitats, as well as their thermal preferences. While pike generally prefers structurally complex habitats and can utilize deeper, colder waters [33, 34], catfish show a high degree of plasticity in both habitat use and favoring warmer conditions [32, 45]. Understanding how these factors contribute to their spatial and trophic interactions is essential for assessing potential competition and its implications for ecosystem structure and predator-prey dynamics.

## Aims

In this study, we built upon on our previous research and tested whether the specific conditions in waterbody can alter the extent of niche overlap of these two predators in terms of their spatial (SNp for pike, SNc for catfish) and trophic (TNp for pike, TNc for catfish) niche overlap (OL). Differences in OL could reflect the different intensity of competition in different waters and have an impact on the individual growth rate, which is a strong indicator of the intensity of competition or the density of available resources [46].

We quantified spatial and trophic niche overlap, as well as growth rates, of pike and catfish in three waterbodies in the Czech Republic, hypothesizing that their overlap is influenced by local environmental conditions such as prey availability and structural complexity. Additionally, we explored whether their use of benthic versus pelagic habitats and their thermal preferences contribute to their niche partitioning.

To achieve this, we employed fine-scale acoustic telemetry to monitor the long-term movements and behaviors of pike and catfish, capturing horizontal and vertical positioning as well as diel habitat use (day vs. night) in benthic vs. pelagic zones. This approach enabled us to assess how these predators might partition space across different times of day and habitat strata. We further quantified their trophic niches using stable isotope analysis (SIA) to determine the degree of dietary overlap.

## Methods

### Study sites

The study was performed in three artificial waterbodies: two oligotrophic man-made lakes, Milada (50° 39′ N, 13° 58′ E) and Most (50° 32′ N, 13° 38′ E), and one eutrophic reservoir, Rimov Reservoir (48°50’N, 14°29’E; Table 1), all located in the Czech Republic. Milada and Most are recently created post-mining lakes resulting from aquatic restorations of mining pits. Aquatic restoration took place from 2001 to 2010 in Milada and from 2008 to 2014 in Most. The Rimov was built in 1978 by damming the Malse River. These waterbodies differ in various environmental conditions, including trophic status, turbidity, structural complexity, and prey availability (Table 1). Further detailed information can be found in [28, 29, 34].

**Table 1:** Environmental parameters of the three waterbodies during telemetry tracking periods. TP - total phosphorus concentration in the water, SD - Secchi depth, status of Littoral structural complexity - see [34, 54], TD - mean depth of thermocline, Gillnet fish CPUE – represents the density of prey fish caught with gillnets, weighted by the volume of habitats sampled with gillnets (see [43])

	Area	Volume	Max. depth	Mean depth	TP	SD	Structural complexity	Stratification*		TD	Gillnet prey fish density	
	(ha)	(10^6^ m^3^)	(m)	(m)	(µg L^− 1^)	(m)		Oxygen	Temperature	(m)	NPUE (ind./1000 m^2^)	WPUE kg/1000 m^2^)
Milada	250	36	25	16	5	5	high	yes	yes	8.3	33	3.9
Most	311	70	75	22	2.5	7.7	low	no	yes	7.9	103	13.6
Rimov	210	34	45	16	26.4	1.7	low	yes	yes	5.5	831	42

* for further information on the presence of oxygen and temperature stratification in the three waterbodies, see [29, 34] and Supplemetary materials (Sect. 1)

Our previous research has shown that both northern pike and catfish adjust their movement, activity, and foraging strategies in response to these local factors in these waterbodies. In Most, pike exhibited larger home ranges, greater use of open waters, a broader depth range, and higher overall activity compared to Milada. These differences were attributed to lower structural complexity in Most, which apparently causes the pike higher activity and space use [34]. In Milada and Most lakes, catfish displayed larger home ranges and higher diurnal activity compared to those in the Rimov Reservoir; we concluded that these variations are driven by differences in prey availability and distribution ( [43]; see more information about prey availability in the Supplementary materials, Sect. 1). Stable isotope analyses further revealed that catfish consistently occupied a broader trophic niche than pike in all three systems [44]. In the Rimov Reservoir, fish constituted the most important prey for both predators, and a similar pattern was observed for pike in Lakes Most and Milada. In Most, catfish also consumed significant amounts of birds and other semi-aquatic prey, whereas in Milada, crayfish formed an important part of their diet [28, 43, 44].

### Fish telemetry

A total of 90 individuals (15 pike and 15 catfish in each waterbody) were caught and tagged for the study. Individuals were measured and weighed prior to tagging (Table 2). Acoustic transmitters (Lotek Wireless Inc, MM-M-11-28-PM, 65 × 28 mm, weight in air of 13 g) were implanted as described in detail in [29, 34]. These transmitters included pressure (all waterbodies), motion (Milada and Most) and temperature (Rimov) sensors. The burst rate was set to 25 s in Milada and Most, and 15 s in Rimov. The fish were caught and tagged between 5 and 10 May 2015 in Milada and Most, and between 19 and 20 April 2017 in Rimov.

**Table 2:** Summary statistics of European catfish (*Silurus glanis*) and Northern Pike (*Esox lucius*) body size across three study lakes (Milada, most, and Rimov). The table presents the number of individuals (N), minimum (Min), mean (Mean), and maximum (Max) values for both weight (kg) and total length (TL, cm). Data are provided separately for individuals tracked using acoustic telemetry and those analyzed through stable isotope analysis (SIA)

		Lake	N	Weight (kg)			TL (cm)		
				Min	Mean	Max	Min	Mean	Max
Telemetry	catfish	Milada	15	3.5	10.7	23.5	81	117.9	158
		Most	15	2.1	5	8.4	71	93.3	115
		Rimov	15	3.4	11.7	35	81	116.6	166
	pike	Milada	12	0.8	4.1	14.2	52	78.9	115
		Most	13	1.5	1.8	7.5	64	86.3	104
		Rimov	13	0.8	2.6	10.9	50	67.6	116
SIA	catfish	Milada	64	2	7.9	13.8	68	104.8	132
		Most	64	2.4	5.1	14.5	80	93.3	138
		Rimov	60	3.7	10.6	21.8	83	111.1	150
	pike	Milada	56	0.7	5.1	10.6	47	86	117
		Most	48	0.6	4.7	7.5	45	74.3	92
		Rimov	60	0.7	3.4	13.8	47	69.3	120

Three separate MAP positioning systems (Lotek Wireless Inc., Canada) were deployed in all waterbodies to track the tagged fish. Each system consisted of tens of receivers (Lotek Wireless Inc., WHS3250; 44, 47 and 91 receivers in Milada, Most and Rimov, respectively) deployed in arrays (array deployment is given in the Supplementary materials, Sect. 2). A detailed description of the design, deployment and accuracy of the arrays can be found for Milada and Most in [34] and for Rimov in [29]. For the purpose of this study, tracking records of summer period June – September (year 2015 in Milada and Most, 2017 in Rimov) were selected to match data collected for stable isotopes (see below).

### Temperature monitoring

To test whether pike and catfish utilize similar habitats, we calculated their presence relative to the thermocline. Temperature stratification was monitored using 60 HOBO Pendant temp/light 64 K data loggers (Onset, USA). In Lakes Most and Milada, loggers were attached at 1-m intervals from the surface to 13 m depth (with an extra logger at 20 m) along a rope secured to a floating buoy anchored at 22 m depth, with deployments at two sites per lake (covering east/west or south/north gradients). In Rimov Reservoir, loggers were deployed at four locations along the reservoir’s longitudinal axis, with units attached at 1‐m intervals from the surface to 13 m depth (plus an additional logger at 20 m in the dam and middle sections). In all waterbodies, data were recorded at 5‐minute intervals, providing high spatiotemporal resolution. The thermocline depth was calculated using the thermo.depth function in the R package rLakeAnalyzer [47]. Further details are provided in [29, 34] and in the Supplementary materials (Sect. 3).

### Stable isotopes

Stable isotopes are commonly used in studies of trophic niche width and species overlap because they capture a broader time window of foraging behaviour compared to traditional stomach content analysis [48, 49]. Here, we used stable carbon (δ^13^C) and nitrogen (δ^15^N) isotopes to determine the overlap of trophic niches. The data used for this study were processed for the study of [44] to determine the total and individual trophic niche width and the degree of individual trophic specialization. A detailed description of tissue sampling, processing and stable isotope analysis can be found in [44]. In brief, in 2017, 15 adult individuals of each species were caught by electrofishing, in Most and Milada in September and in Rimov in July. All individuals were measured and weighted. Samples of four body tissues (muscle, fin, plasma, and blood) were collected, but for this study stable isotope signals from the dorsal muscle (biopsy punch, skin removed) were used only, as they are the most representative for determining the trophic niche [50]. All samples were dried (at 60 °C for 48 h) and ground into a homogeneous powder. All SIAs were performed using a FlashEA 1112 elemental analyser coupled to a Thermo Finnigan DELTAplus Advantage mass spectrometer (Thermo Fisher Scientific Corporation, Waltham, MA, USA) at the University of Jyväskylä, Finland. The isotope ratios of carbon and nitrogen are reported as δ^13^C and δ^15^N, relative to the international standards for carbon (Vienna PeeDeeBelemnite, Vienna, Austria) and nitrogen (atmospheric nitrogen).

Since δ^15^N and δ^13^C for basal resources can vary considerably among sites, the results from SIA were corrected to baseline. Trophic positions of fish were calculated following [51], using δ¹⁵N values of primary consumers (zebra mussels, aquatic snails, and water lice) as a baseline. To account for site-specific variation in basal resources, δ¹³C values were corrected according to [52] using the mean δ¹³C of primary consumers and their isotopic range. The resulting values include trophic position (TP) based on δ¹⁵N and baseline-corrected δ¹³C (δ¹³C_corrected_). A detailed description of the correction method is provided in [44].

### Growth

The data on pike growth used in this study were partly processed for the study by [34], which compared pike growth from Milada and Most. Here, we expanded this dataset by incorporating new data from Rimov, processed using the same methodology as in [34]. Age determination and growth calculation for pike were conducted using scale reading, with three scales analyzed per individual. The results were averaged and used to back-calculate size-at-age using the Fraser–Lee equation [53]. Since scales were only available for the pike tracked via telemetry, we focused on body growth increment during the year prior to tagging, as this best reflects individual behavior and movement patterns during the telemetry study.

For catfish, we included previously published growth data from all three waterbodies [43] to enable comparisons with pike. These long-term monitoring data were collected as part of ongoing research [43]. During these monitoring efforts, all captured catfish were tagged with Passive Integrated Transponders (PIT tags) and subsequently released back into their respective lakes. Growth was assessed through recaptures, calculated as the difference in total length between initial tagging and the most recent recapture, scaled per year (length gain/year). These data were collected between 2013 and 2017 in Milada and Most, and 2017–2019 in Rimov Reservoir. Only individuals with a minimum recapture interval of one year were included to avoid short-term fluctuations. Since catfish growth data were already published, they were not reanalyzed in this study but were included for comparative purposes to evaluate differences in growth patterns between pike and catfish across the same waterbodies. Despite differences in aging methods between species, our approach ensures comparability of growth estimates by focusing on recent body growth increments rather than full life history trajectories.

### Data analysis

#### Telemetry data

The locations of the individual fish were calculated using the proprietary post-processing software UMAP v.1.4.3 (Lotek Wireless Inc., Newmarket, Ontario, Canada). The filtering and further post-processing of the fish locations are described in detail in [34] and in Supplementary material (Sect. 2). The filtered fish locations, which included all three dimensions (latitude, longitude and depth), were then matched with the bathymetry maps of each waterbody (with a grid resolution of 1 × 1 m). The bottom depth was determined at each fish location.

To quantify habitat use, we calculated the proportion of time each individual spent in different habitats considering benthic and open water areas as well as position of thermocline. Habitats were defined by two criteria: vertical distance from the bottom and position relative to the thermocline. Areas less than 5 m from the bottom were categorised as “benthic”,” while areas 5 m or more from the bottom were considered “open water”.” In addition, each of these groups was subdivided according to whether the individual was above or below the thermocline (mean depth for each day), resulting in four different habitat types (herein reffered as Benthic Above, Benthic Below, OW Above, OW Below). The proportions of habitat utilization were calculated separately for each individual, diel period (day and night), and each day to capture temporal variations in habitat preferences.

To analyze spatial niche overlap, fish depth and bottom depth at the fish location were used as two meaningful niche axes to analyze fish spatial niche. Depth was considered as a vertical axis and bottom depth as a horizontal axis to account for differences between benthic and open water areas [54, 55].

### Habitat use

To analyze habitat use in relation to species, waterbody, diel period, and their interactions, we applied a generalized linear mixed-effects model (GLMM) using the ‘glmmTMB’ package [56]. Specifically, we modeled the proportion of time spent in each habitat as a function of habitat type, species, waterbody, diel period, and their interactions (full factorial interaction), while accounting for repeated measures within individuals by including as a random intercept effect. We fitted the model using a beta regression to appropriately handle proportional response data (values between 0 and 1). Their full factorial interaction allowed us to assess how habitat use patterns varied across species, waterbody, and diel periods. Model selection and validation included checking for residual patterns, overdispersion, and the goodness-of-fit using diagnostic plots. To visualize the modeled habitat use across different conditions, we used the plot_model() function from the sjPlot package [57]. This approach allowed us to generate predicted values from our GLMM, accounting for key interactions among habitat, species, waterbody, and diel period. The predictions were computed at moderate values of continuous covariates to standardize the visualization. To evaluate species-specific habitat preferences across different lakes and diel periods, we performed pairwise contrasts using the emmeans package [58]. Our contrast selection focused on three key comparisons: (i) preferred habitat of a species in each waterbody; (ii) interspecies comparison in the same habitat within each waterbody; (iii) comparison of a species’ habitat use among waterbodies.

### Spatial and trophic niche overlap

To visualize the overlap of spatial (OL-SN) and trophic niche (OL-TN) between pike and catfish, we used the R package rKIN [59], which calculates the kernel utilization distributions (UD) for spatial and trophic niches. Kernel UD effectively reflects the asymmetric nature of fish distribution and provides a more realistic overlap than the commonly used ellipses. However, rKIN does not determine the uncertainty in the probability of overlap between species. To address this issue, we used the R package nicheROVER [60].

We created spatial (SN) and trophic (TN) niche regions for each species-waterbody combination as Bayesian ellipses, with 95% ellipse size. Thus, the niche region of a species (SNp, SNc, TNp, TNc) is the area in which a random pike (p_i_) or random catfish (c_i_) occurs with 95% probability. The probabilities of spatial overlap were calculated using a Monte Carlo algorithm with ten thousand independent draws from the distributions of catfish and pike. The overlaps were determined according to how many of these draws fell within the spatial or trophic niches of the other species. The overlaps were expressed as the probability that a random pike would be found in the spatial niche of a catfish and vice versa: OL-SNp/c = Pr(p_i_ ∈ SNc) and OL-SN(c/p = Pr(c_i_ ∈ SNp). For trophic niche overlaps, the expressions would be the same, but labeled OL-TN and TN_i_. These overlaps are directional, so for example OL-SN(p/c) is not the same as OL-SN(c/p), which is consistent with [60].

To test differences in spatial and trophic overlap between species and waterbodies, Monte Carlo estimate probabilities were then compared using generalized linear model (GLM) with a Gaussian distribution and an identity link function, which is suitable for continuous response variables. The model estimated the probability of OL-SN as a function of species overlap (OL-SN(p/c) or OL-SN(c/p)), waterbody, diel period, and their interactions. For OL-TN, we applied a similar Gaussian GLM without diel period, as it was not relevant to isotopic data. The model assessed trophic overlap (OL-TN) based on species overlap (OL-TN(p/c) or OL-TN(c/p)), and waterbody. All models were fitted using the logit link function, and statistical significance was assessed through Wald tests and confidence intervals. We also plotted the trophic niche width for both species to provide a complementary comparison. Previous analyses of trophic niches of the two species showed that they were broader for catfish in all studied waterbodies [44].

### Growth

We used general linear models to find differences in individual growth of pike in the previous season (body growth; dependent variable) among waterbodies, using waterbody identity and body length of fish as explanatory variables. As the distribution of the response variables was positively skewed and continuous, we assumed a gamma distribution with a log link function to account for the nature of the data. We initially tested a model including the interaction term between waterbody and TL (increment ~ Lake * body length), but this did not significantly improve model fit compared to the additive model (*increment ~ Lake + body length*). Therefore, we proceeded with the additive model, which provided a more parsimonious fit while still accounting for potential differences in growth among lakes. The calculations of the predicted growth values for each waterbody and the pairwise comparison of growth were carried out with the emmeans package [58].

We also plotted differences in individual growth of catfish to provide a complementary comparison. Previous analyses of catfish growth showed that catfish growth in terms of annual body increment was waterbody dependent [43]. Data processing and statistical analysis were performed using R software version 4.4.1 [61].

During the preparation of this work the authors used Large Language Models ChatGPT-4o in order to refine and correct language of the text. After using this tool, the authors reviewed and edited the content as needed and take full responsibility for the content of the publication.

## Results

### Habitat use

**Fig. 1:**
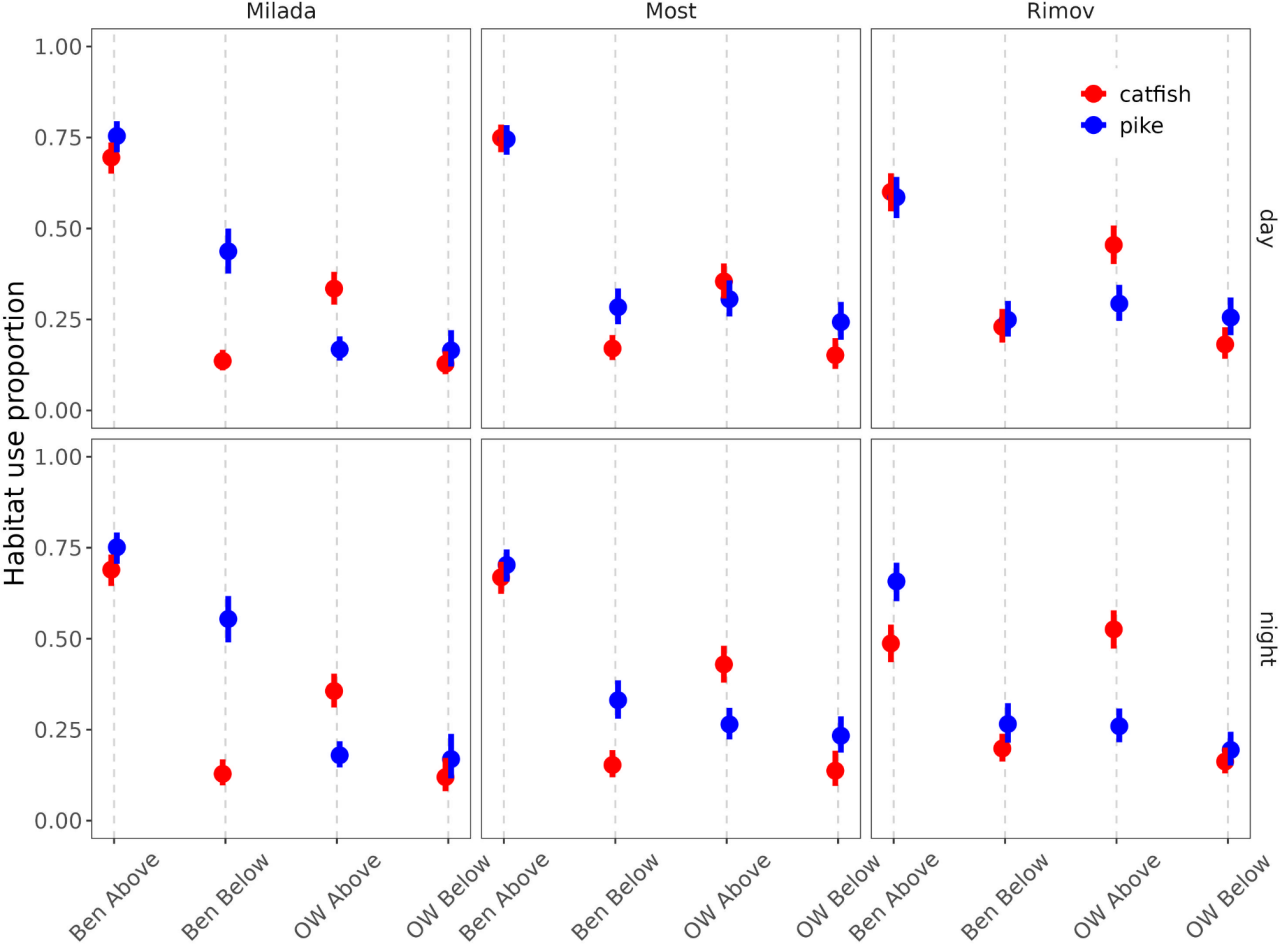
Model-predicted habitat use proportions for catfish and pike across three waterbodies (Milada, Most, and Rimov) during daytime (top row) and nighttime (bottom row). The x-axis represents different habitat types: Ben Above (benthic above the thermocline), Ben Below (benthic below the thermocline), OW Above (open water above the thermocline), and OW Below (open water below the thermocline). Points represent model predictions, while error bars indicate 95% confidence intervals

**Fig. 2:**
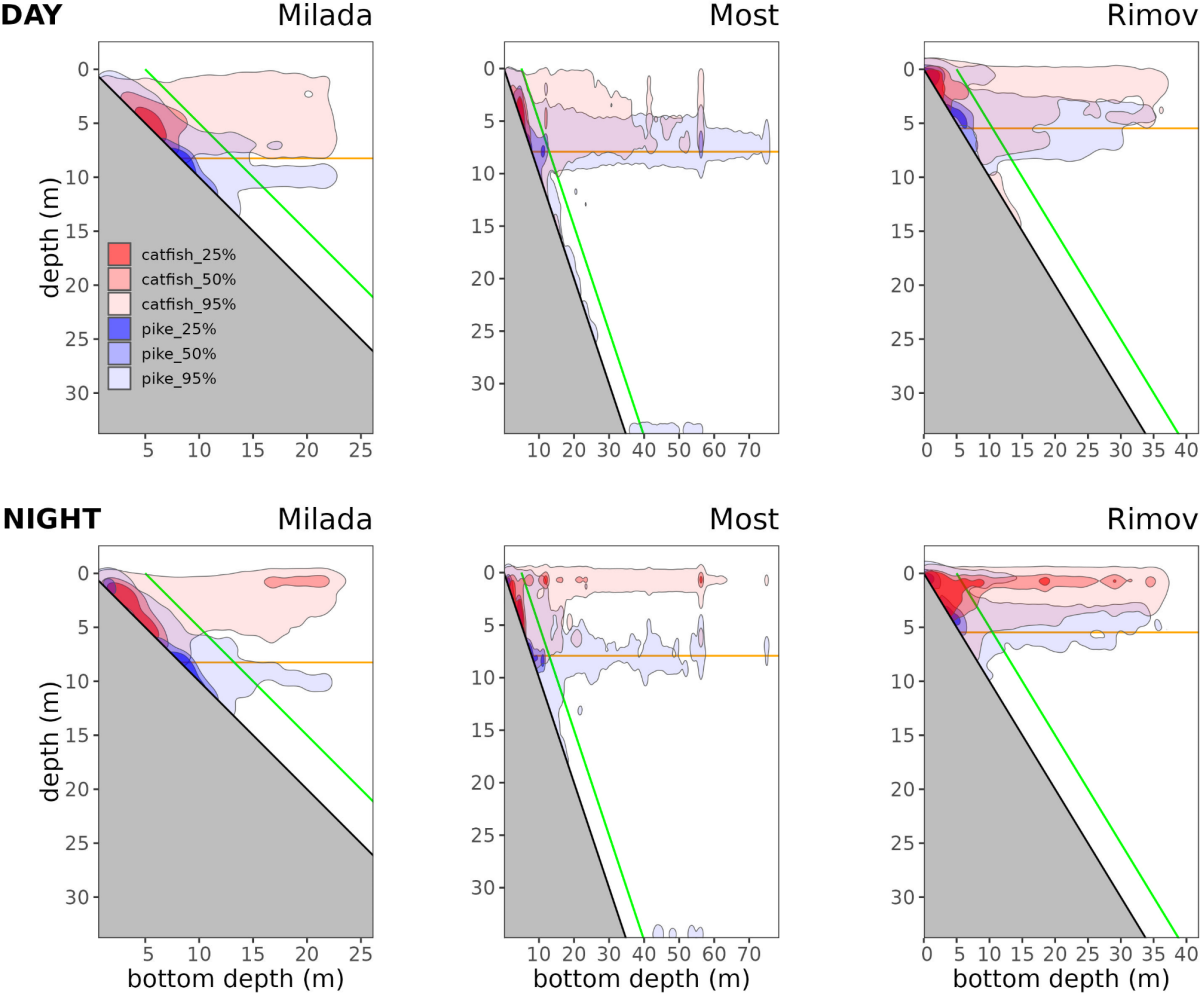
Kernel utilization distributions (25%, 50%, and 95%) of depth and bottom depth use by catfish (red) and pike (blue) across three waterbodies (Milada, Most, and Rimov) during daytime (top row) and nighttime (bottom row). The x-axis represents bottom depth (m), while the y-axis represents water column depth (m). The orange line represents the thermocline, and the green line denotes the boundary used to separate benthic and open water habitats

During the day, both pike and catfish primarily occupied benthic habitats above the thermocline, while their use of alternative habitats, benthic below, open water above, and open water below, was significantly lower (Figs. 1 and 2, detail results are given in Supplementary materials Sect. 4). Despite this overall pattern, species-specific differences emerged. In most cases, pike used open water above the thermocline less frequently than catfish, while showing a greater tendency to occupy benthic and open water habitats below the thermocline. Conversely, catfish exhibited higher use of open water above the thermocline and lower use of both benthic and open water habitats below it. However, the magnitude and significance of these differences varied across waterbodies (Fig. 1, SM Sect. 4). For example, pike demonstrated greater use of benthic habitats below the thermocline in Milada and higher use of open water above the thermocline in Most and Rimov. Meanwhile, catfish showed a preference for benthic habitat above the thermocline in Milada and Most, while its use of open water above the thermocline was lower in these lakes, though this difference was not statistically significant (SM Sect. 4).

At night, the overall habitat-use pattern remained largely consistent, with benthic habitats above the thermocline continuing to be the dominant habitat for both predators (Fig. 1 and 2, SM Sect. 4). Diurnal changes in habitat use were generally subtle, but some diel differences emerged, even though none were statistically significant (SM Sect. 4). Across all waterbodies, pike generally showed greater use of benthic habitats below the thermocline, while catfish increased use of open water above the thermocline. However, the magnitude of these differences varied among waterbodies (Fig. 1, SM Sect. 4), while maintaining a similar waterbody- and species-specific pattern as observed during the daytime.

### Spatial niche overlap and width

The habitat-use patterns described above were reflected in spatial niche overlap depending on the waterbody, time of day and direction of species interaction. Across all waterbodies, pike exhibited a higher spatial overlap with catfish than the reverse, Most and Rimov had higher overall spatial overlap between species, while Milada exhibited the lowest and overlap between species was higher during day time (Table 3).

**Table 3:** Results of the generalized linear model (GLM) testing the dependence of Spatial overlap extent on species, waterbody, and diel period, including their interactions. Odds ratios represent the likelihood of Spatial overlap between Pike and catfish across different lakes and diel periods. CI = 95% confidence interval; p = significance value. Significant results (*p* < 0.05) are in bold

Predictors	Odds Ratios	CI	*p*
(Intercept)	0.51	0.51–0.51	**< 0.001**
species_int. [pike_catfish]	0.2	0.20–0.20	**< 0.001**
waterbody (wb) [Most]	0.48	0.48–0.49	**< 0.001**
waterbody (wb) [Rimov]	0.37	0.37–0.37	**< 0.001**
diel period (dp) [night]	-0.16	-0.16 – -0.16	**< 0.001**
species_int. [pike_catfish] × wb [Most]	-0.72	-0.72 – -0.72	**< 0.001**
species_int.[pike_catfish] × wb [Rimov]	-0.19	-0.19 – -0.19	**< 0.001**
species_int.[pike_catfish] × dp [night]	-0.03	-0.03 – -0.03	**< 0.001**
wb [Most] × dp [night]	-0.12	-0.12 – -0.12	**< 0.001**
wb [Rimov] × dp [night]	-0.06	-0.06 – -0.06	**< 0.001**
(species_int. [pike_catfish] × wb [Most]) × dp [night]	0.38	0.38–0.38	**< 0.001**
(species_int. [pike_catfish] × wb [Rimov]) × dp [night]	0.2	0.20–0.20	**< 0.001**

**Fig. 3:**
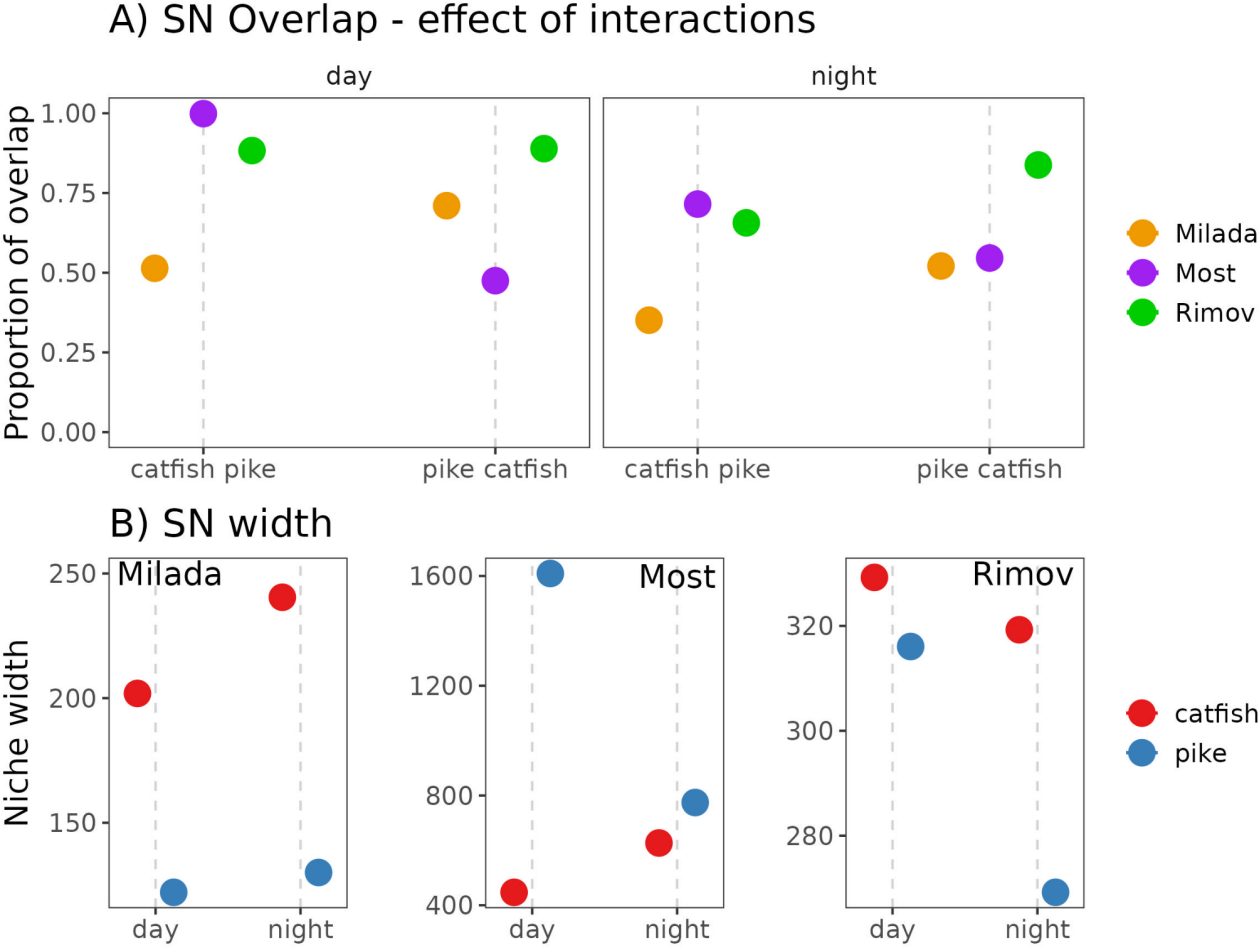
Spatial niche overlap and spatial niche width of catfish and pike across three waterbodies (Milada, Most, and Rimov) during daytime and nighttime. (**A**) Proportion of spatial niche (SN) overlap between catfish and pike, with values closer to 1 indicating higher overlap. Colors represent different lakes. (**B**) SN width (niche width) of catfish (red) and pike (blue) in each lake, showing diel differences in niche breadth. Dots represent model-predicted values

However, local conditions and diel period inractions played important role (Fig. 3A). Regarding variation among waterbodies, during both diel periods, catfish exhibited greater spatial overlap with pike in Most and Rimov than in Milada (Fig. 3A). While pike had higher spatial overlap with catfish in Rimov than in Milada and Most (Fig. 3A). Regarding within-waterbody variation, in Milada, catfish had lower spatial overlap with pike than vice versa. In Most, the opposite was observed, with catfish exhibiting higher spatial overlap with pike than vice versa. In Rimov, overlap was similar in both directions during the day, but at night, catfish had lower overlap with pike than vice versa (Fig. 3A).

Spatial niche width varied across species, diel periods, and waterbodies (Fig. 3B; Table 4). In Milada, catfish exhibited signifficatly broader niche width than pike, particularly at night when their niche expanded further. In contrast, pike maintained a relatively narrow niche width throughout both diel periods, with only a slight signifficant increase at night (Fig. 3B; Table 4). In Most, the pattern was reversed: pike displayed a significantly broader niche width than catfish, especially during the day. At night, pike’s niche width significantly decreased while catfish’s increased, reducing the difference between them (Fig. 3B; Table 4). In Rimov, catfish consistently had a significantly broader niche width than pike during both day and night. However, this difference was more pronounced at night, as the niche width of both species significantly decreased—though the reduction was greater for pike (Fig. 3B; Table 4).

**Table 4:** Results of the generalized linear models (GLMs) testing the effects of species (pike vs. catfish), diel period (day vs. night), and their interaction on Spatial niche width in each waterbody (Milada, most, and Rimov) separately. The response variable (niche width) was log-transformed for analysis. Estimates (Est.) represent model coefficients, with 95% confidence intervals (CI) and associated p-values (p). Significant effects (*p* < 0.05) indicate differences in Spatial niche width between species and diel periods. Interaction terms assess whether diel effects on Spatial niche area differ between species within each lake

*Predictors*	Milada			Most			Rimov		
	*Est.*	*CI*	*P*	*Est.*	*CI*	*p*	*Est.*	*CI*	*p*
(Intercept)	5.31	5.31–5.31	**< 0.001**	6.1	6.10–6.10	**< 0.001**	5.8	5.80–5.80	**< 0.001**
species [pike]	-0.5	-0.50 – -0.50	**< 0.001**	1.28	1.28–1.28	**< 0.001**	-0.04	-0.04 – -0.04	**< 0.001**
diel period (dp) [night]	0.17	0.17–0.17	**< 0.001**	0.34	0.34–0.34	**< 0.001**	-0.03	-0.03 – -0.03	**< 0.001**
species [pike] × dp [night]	-0.11	-0.11 – -0.11	**< 0.001**	-1.07	-1.07 – -1.07	**< 0.001**	-0.13	-0.13 – -0.13	**< 0.001**

### Trophic niche overlap and width

**Fig. 4:**
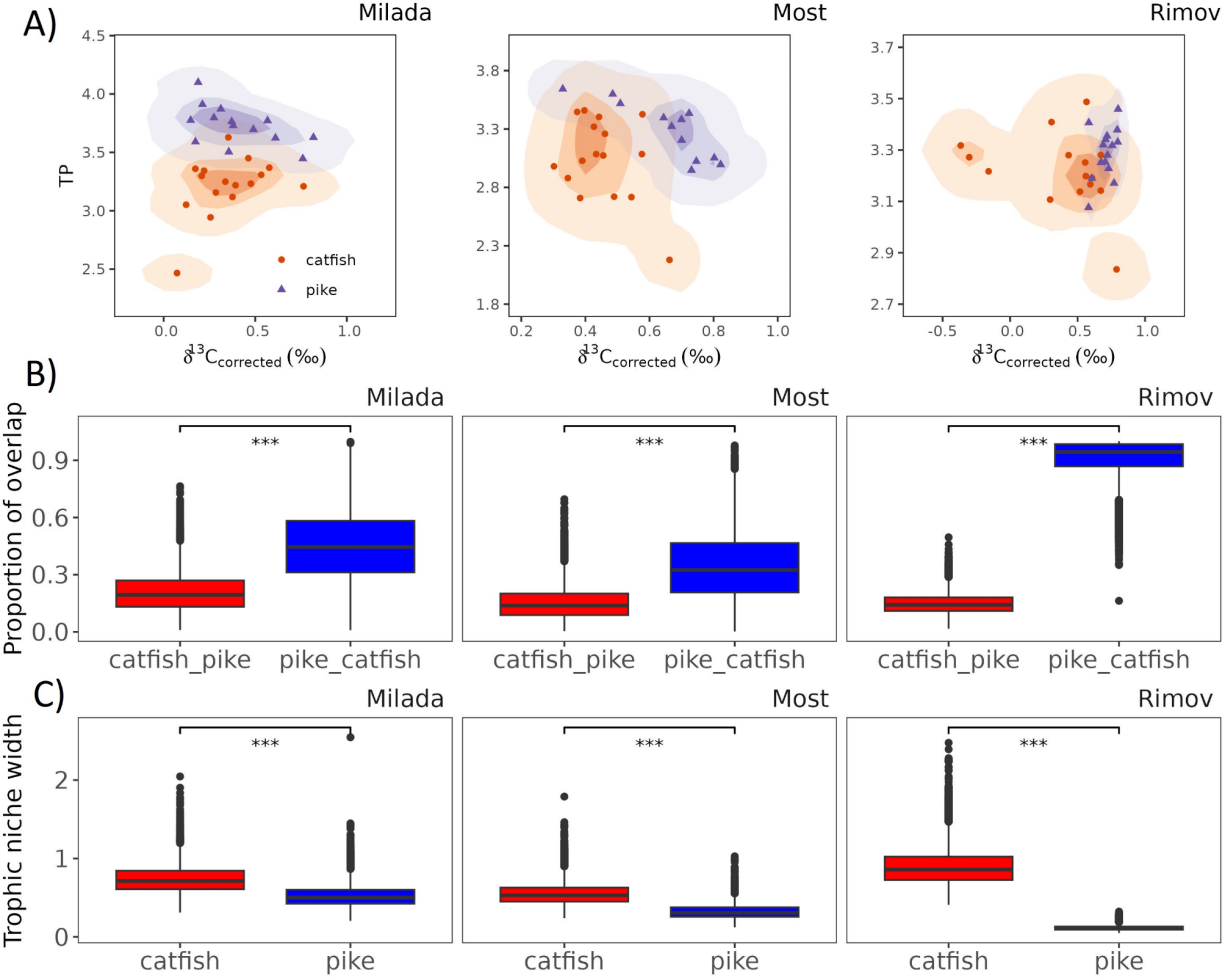
Trophic niche overlap and trophic niche width of catfish and pike across across three waterbodies (Milada, Most, and Rimov). (**A**) Trophic niche overlap based on stable isotope values of δ¹³C_corrected_ (‰) and trophic position (TP), with kernel density estimates (25%, 50%, and 95%) representing species’ isotopic niches. (**B**) Extent of trophic niche overlap between catfish (red) and pike (blue), showing the proportion of shared isotopic space. (**C**) Trophic niche width, comparing the individual variation in isotopic space used by each species. Asterisks (* *p* < 0.05, ** < 0.01, *** < 0.001) indicate statistically significant differences between species

Trophic niche overlap varied from 0.15 to 0.91 across waterbodies, with pike showing a significantly greater overlap (0.35–0.91) than catfish (0.15–0.21; *p* < 0.001; Fig. 4A, B). The largest difference between pike overlap with catfish thn catfish overlap with pike was observed in Rimov (Least square mean on the mean difference (mean, ± SE): -0.76 ± 0.002, *p* = 0.001), followed by Milada (-0.242 ± 0.02, *p* = 0.001) and Most (-0.192 ± 0.00, *p* = 0.001) (Fig. 4B). In Milada, pike had higher TP values, but a similar δ^13^C_corrected_ range to catfish (Table 5). In Rimov and Most, the TP ranges were similar for both species, but the pike had higher δ^13^C_corrected_ values (Table 5). The width of the trophic niches was different in all waterbodies. However, catfish had the widest trophic niche in all waterbodies, with the greatest difference between catfish and pike in Rimov (Fig. 4C; Vejřík et al. 2023).

**Table 5:** Mean value ± standard deviation of baseline corrected the stable isotope signals of δ^13^C_corrected_ and TP for both species in studied waterbodies

Waterbody	Species	Mean ± SD δ13C_cor_. (‰)	Mean ± SD TP
Milada	catfish	0.35 ± 0.18	3 ± 0.4
	pike	0.40 ± 0.22	3.3 ± 0.2
Most	catfish	0.45 ± 0.10	3.2 ± 0.3
	pike	0.66 ± 0.14	3.7 ± 0.2
Rimov	catfish	0.38 ± 0.37	3.2 ± 0.1
	pike	0.71 ± 0.07	3.3 ± 0.1

### Growth

**Fig. 5:**
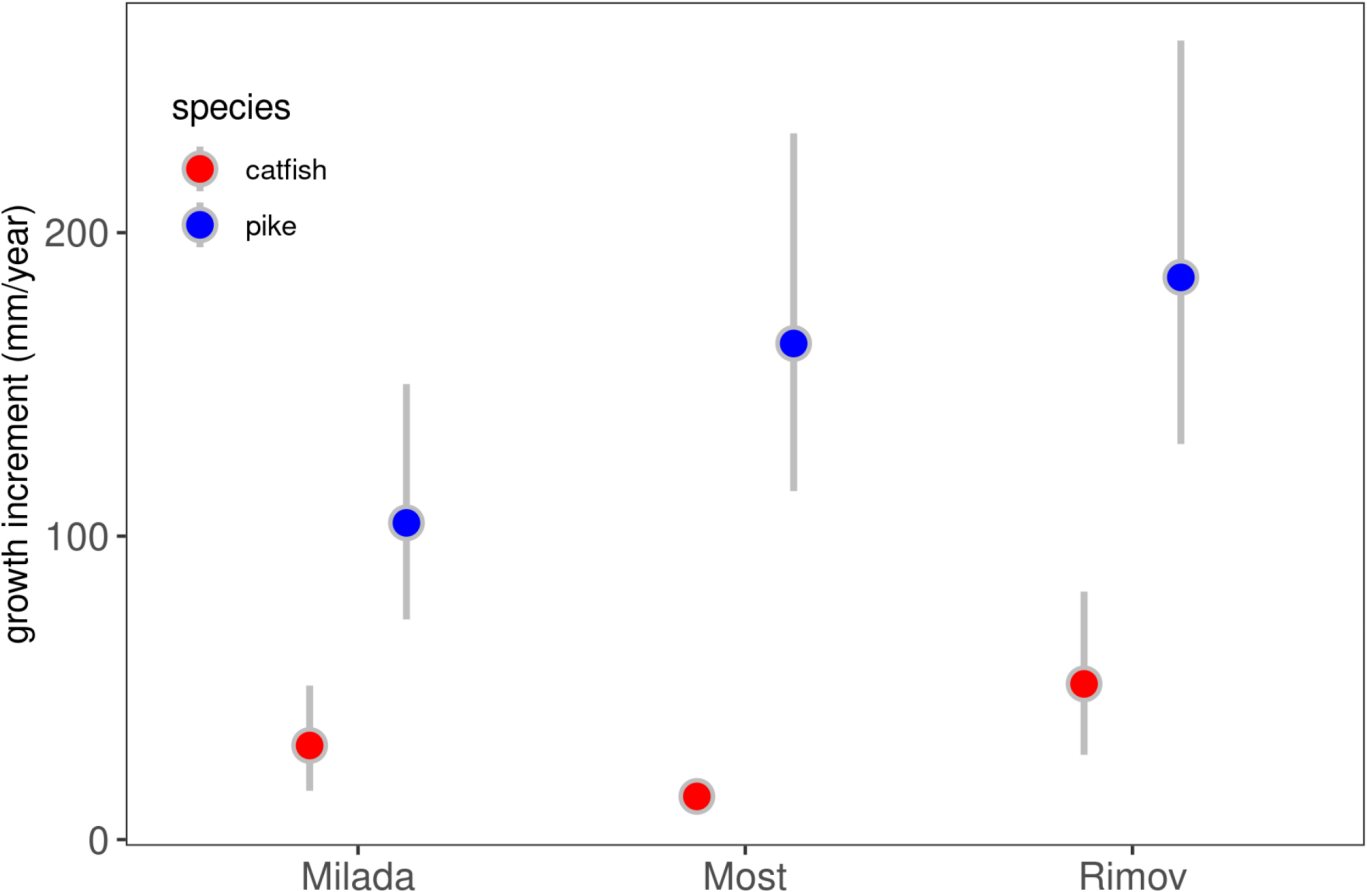
Growth (in mm/year) of pike and catfish across across three waterbodies (Milada, Most, and Rimov). Growth of catfish according to [43]. The dots show the estimated mean values from the GLM model and the 95% confidence intervals of the mean values (whiskers)

Pike growth was significantly higher in Rimov and Most compared to Milada, with significant differences between Milada and Rimov (Least square mean on the mean difference (mean, ± SE): -0.574 ± 0.178, *p* = 0.007) and between Milada and Most (-0.449 ± 0.171, *p* = 0.032). Growth rates in Most and Rimov were statistically similar (-0.125 ± 0.180, *p* = 0.768). Body length had a significant effect on growth (estimate ± SE = -0.001 ± 0.0004, *p* = 0.03). The selected model did not include an interaction term between waterbody and TL, as it did not improve model fit. Thus, the reported growth estimates of pike represent the mean predicted values across individuals of average total length (TL) of 77.8 cm (Fig. 5). We also plotted differences in individual growth of catfish to provide a complementary comparison with pike. As previously reported, catfish growth in terms of annual body increment varied among waterbodies (see Methods for details on data source; Fig. 5). The reported growth estimates of catfish represent the mean predicted values across individuals of average total length (TL) of 90.1 cm (Fig. 5).

## Discussion

Our study revealed that during summer, pike and catfish predominantly occupied benthic habitats above the thermocline across all studied waterbodies and diel periods. However, species-specific differences emerged, with catfish making greater use of open water above the thermocline and pike utilizing both open water and benthic habitats below it more frequently. These habitat use patterns varied across waterbodies, with subtle diel shifts that did not reach statistical significance. Despite these variations, the two species exhibited substantial spatial overlap, though the degree of overlap fluctuated across waterbodies and diel periods. Spatial niche width also differed among waterbodies, with catfish occupying a broader spatial niche in two waterbodies, while pike had a broader niche in one waterbody. In contrast, trophic niche overlap was significantly greater for pike with catfish than vice versa, with pike almost completely overlapping with catfish in one of the waterbodies studied.

The depth-related spatial differentiation between pike and catfish aligns with thermal preferences, which likely play a key role in shaping their habitat selection. Both species differ in their thermal optimum for pike 10–24 °C and 12–28 °C for catfish [62], with temperature preferences ranging between 16 and 21 °C for adult pike [63] and 26–28 °C for catfish [64]. These differences suggest that temperature could be a important factor driving habitat partitioning between the two species. Consistent with these thermal preferences, previous research has shown that in open water pike frequently stay near the thermocline [34, 45, 65], where temperatures remain within their preferred range. In contrast, catfish favor the warmer epilimnion and actively avoid colder meta- and hypolimnetic waters [29, 45]. Our findings strongly support these established patterns. Pike demonstrated a higher capacity to utilize habitats below the thermocline, whereas catfish remained largely restricted to the water layers above thermocline. Interestingly, water temperatures below the thermocline were consistent across all studied water bodies (8–9 °C; see Supplemetraty materials, Sect. 3), suggesting that pike can tolerate a broad thermal range and may exhibit greater ecological flexibility in stratified system.

The pike is considered a preferentially littoral-bound predator [66], while the catfish tends to rest in the littoral and forages in the open water [26]. Our study highlights that a shallow benthic habitat is essential for both species across diel periods. However, pike was more strongly associated with benthic habitats than catfish, which utilized open water more extensively. This resulted in substantial spatial overlap, though the extent of partitioning varied by waterbody. Local environmental factors such as prey availability and structural complexity as well as diel space use pattern likely shape these habitat use patterns, ultimately influencing the degree of spatial segregation between these two predators.

Prey distribution and availability are key drivers of predator habitat use, potentially shaping the degree of niche partitioning across different water bodies. Since prey distribution is often dynamic, it can lead to shifts in predator spatial patterns, influencing both inter- and intraspecific interactions [67]. The distribution of fish prey varied among waterbodies to some extent and it could modify spatial distrubiton of predators as well as their overlap. In Rimov, fish prey was found almost exclusively in benthic and open water areas above the thermocline, a pattern also observed in Milada, where prey fish layers were also more concentrated just below the thermocline in benthic habitats [34, 43] (Supplementray materials, Sect. 1). In Most, the fish prey is densest in the epilimnion and shallow nearshore, but cold-water coregonids are found in the deep benthic and in hypolimnetic areas [34]. These distributions align well with our results on pike and catfish space use: both predators predominantly remain above the thermocline in bethic or open water habitat where prey is most abundant, but pike descends to exploit prey in the deeper layers in Milada and Most. It is supported by our previous finding from Most, where coregonids were found in pike stomachs [28], and they are the only fish species present in benthic and pelagic areas below 12 m depth [34]. Pike’s ability to access deeper prey in some waterbodies likely contributes to moderate niche partitioning and reduced direct competition with catfish.

However, prey distribution alone does not fully explain the observed vertical stratification in open water. In addition to foraging opportunities, other ecological and physiological factors may be influencing pike’s habitat use. Across waterbodies, catfish were concentrated near the surface, while pike consistently remained closer to the thermocline (Fig. 2). This pattern is particularly intriguing because, aside from Most, prey availability around the thermocline in open water is relatively low (see Supplementray materials, Sect. 1). In Milada and Rimov, prey fish are concentrated near the surface, aligning closely with the distribution of catfish, while pike’s presence near the thermocline suggests that factors beyond prey distribution are at play. Several possible explanations exist, including thermoregulation [63], allowing pike to remain in energetically favorable conditions. Additionally, light availability and ambush efficiency may play a role—pike, as a visual predator [68], may find the dimmer light conditions around the thermocline more suitable for hunting, compared to the well-lit surface, where prey might detect and evade them more easily [69, 70]. In conclusion, this dual mechanism of spatial partitioning—with catfish primarily utilizing surface waters and pike occupying deeper strata—likely contributes to a reduction in direct spatial overlap, particularly in Milada and Most.

Beyond prey availability, structural complexity may also influence spatial overlap between these predators. Our previous study [34] found that lower complexity in Most contributed to pike’s larger space use and greater reliance on open water. The findings here support that conclusion, as pike also exhibited high open water use in Rimov, which, like Most, has minimal structural complexity, lacking macrophytes and submerged structures [54]. In both Rimov and Most, this likely contributed to greater spatial overlap between catfish and pike, as pike’s increased use of open water led to greater spatial convergence with catfish, which also frequently utilizes this habitat for foraging. In contrast, Milada, which has greater structural complexity, exhibited lower spatial overlap, suggesting that habitat features likely can mediate predator interactions and niche differentiation.

Pike and catfish are often considered to have distinct diurnal activity patterns, with pike being primarily diurnal and catfish nocturnal [32, 68]. However, our results revealed only minor, non-significant diel differences in habitat use and moderate variation in spatial niche overlap and width, leading to slightly lower overlap at night, particularly for catfish with pike. Our study primarily focused on habitat use rather than direct activity levels, but previous research indicates that catfish activity patterns are flexible. Catfish in Milada and Most exhibited higher diurnal activity compared to Rimov [43], likely increasing temporal overlap with pike’s expected activity patterns. This would result in greater temporal overlap with the expected activity pattern of pike in these waterbodies. Although pike activity was not explicitly analyzed in this study, prior research indicates high inter-individual variability in diel activity [69, 71], meaning that some individuals may forage at night, potentially increasing nocturnal overlap with catfish. However, movement and activity patterns were beyond the scope of the study. Overall, our findings suggest that both species exhibit large spatial overlap across diel periods, but further investigation into diel activity patterns is needed to fully understand their temporal interactions.

Our results show that the trophic niche of pike overlaps to a greater extent with catfish niche in all the waters analyzed than vice versa, and that the extent of that overlap is waterbody-dependent. This pattern is not solely a result of catfish’s broader dietary niche but reflects ecological differences in feeding strategies and prey use. These findings can be explained by the greater plasticity and broader range of catfish diets compared to pike as well as the differences in the type of available prey for both species among waterbodies. Despite the ability to utilize a broad food spectrum [26], the diet of pike is more restricted to fish prey than that of catfish [28, 30]. Previous studies have shown that Milada and Most are oligotrophic waters with a strong restriction of prey availability [34, 38]. The pike in these two water bodies forage exclusively on fish [28], while the catfish utilizes a relatively large proportion of non-fish prey such as crayfish in Milada and semi-aquatic prey (waterfowl and mammals) in Most [28, 43]. On the other hand, an important part of both pike and catfish diet consisted of the same species, roach (*Rutilus rutilus*) and European perch (*Perca fluviatilis*) which are dominant species in these lakes [28]. It means that pike shared a part of their diet (fish) with higher intensity. A similar explanation can be proposed for the differences in Rimov. The prey of both predators in the reservoir consisted of fish, as other prey types (crayfish, waterfowl) are scarce and prey fish are more abundant than in Milada and Most [43]. Catfish can prey on a wider range of fish sizes than pike due to their larger size and lower gape limitation [28], e.g. large freshwater bream (*Abramis brama*), which predominate in open waters and are a common prey of catfish (Vejřík L., pers. comm). The pike must specialize on smaller fish prey, which are probably also used by catfish. Overall, our findings indicate that trophic niche overlap is not solely dictated by differences in niche breadth, but rather by the degree of shared resource use, which is influenced by prey availability and species-specific foraging constraints.

Competition is the most important driving force of niche partitioning [14]. Differences in the degree of competition may explain the variation in trophic niche overlap in waterbodies. Higher levels of competition can lead to shifts in the trophic niche, resulting in lower overlap in Milada and Most compared to Rimov. Milada and Most are oligotrophic waters with a limited supply of fish prey. According to Říha et al. [43] Rimov has 8 to 25 times higher prey fish abundance than Most and Milada, respectively. However, the density of predatory fish in these two lakes is higher than in Rimov due to intensive biomanipulation stocking programs [34]. Vejrik et al. [72] calculated the longline CPUE (catch per unit effort) of both predators, which was 0.27, 0.24, and 0.16 per longline snood in Most, Milada, and Rimov, respectively. Therefore, competition for fish prey is higher in Most and Milada. High prey diversification could help to reduce competition in these two waterbodies and mitigate the negative mutual effects between these two predators, thus promoting their coexistence. In Rimov, competition is lower due to higher fish prey density, allowing both species to utilize similar resources without significant negative impacts. This conclusion is strongly supported by the highest body growth of both species in Rimov.

However, beyond prey availability and direct competition between pike and catfish, other ecological factors may contribute to niche plasticity and spatial overlap. The presence of additional piscivorous species and ontogenetic shifts in predator size could also influence the observed patterns, as niche dynamics are shaped by broader community interactions [73, 74]. If other piscivores exert predation pressure or resource competition, this could alter habitat use and trophic niche partitioning between pike and catfish. Additionally, ontogenetic shifts in prey use and habitat selection, commonly observed in predatory fish, may further mediate interspecific interactions [75, 76].While our study focuses on these two species, future research should consider the broader piscivore community and its potential effects on niche plasticity and overlap.

To fully understand the effects of these differences in niche overlap on both species, further studies are needed in a controlled environment where predator presence, prey availability and prey type can be controlled and manipulated. With such a design, we would be able to understand the effects of niche overlap on survival, growth and population density in different contexts. This would not only improve our understanding of ecological interactions, but also provide information for management strategies to maintain balanced fish populations in different aquatic ecosystems.

The invasion of catfish in European waters and their significant niche overlap with native species such as pike may put pressure on native pike populations. Native pike populations may be selected for traits that enhance their competitiveness or reduce overlap with catfish, potentially leading to changes in behavior, spatial use or diet. These dynamics will likely continue to shape outcomes for both species, influencing their ecological roles, competitive strategies, and adaptability in diverse aquatic ecosystems. However, our study suggests that these effects may be strongly influenced by the environmental conditions in the respective invaded waterbody. In addition, both species are used for biomanipulation [77], and our study has shown that combining them in stocking programs can lead to predation pressure in a wide range of trophic resources and in different habitats. However, supporting catfish could be problematic in waters where protected crayfish or birds are present, and where fish prey is scarce, as catfish may primarily target these protected sources and impact their populations [78].

## Conclusions

Our study highlights how environmental variation influences spatial and trophic niche partitioning between pike and catfish, emphasizing the role of habitat structure, prey availability, and behavioral plasticity in shaping predator interactions. These findings demonstrate that niche partitioning is context-dependent, with broad implications for predator-prey dynamics and fisheries management. Furthermore, the high foraging behavior plasticity observed in both species suggests that their interspecific interactions are dynamic, potentially leading to divergent ecological roles over time. Future research should focus on fine-scale movement patterns, prey selection, and competitive interactions under controlled conditions to better understand the mechanisms driving these complex ecological relationships.

## Electronic supplementary material

Below is the link to the electronic supplementary material.

Supplementary Material 1

## Data Availability

Telemetry data will be made available on request, stable isotopes data are available in Vejřík et al. 2023 10.3390/biology12081113.

## References

[1] Petalas C, Lazarus T, Lavoie RA, Elliott KH, Guigueno MF. Foraging niche partitioning in sympatric seabird populations. Sci Rep. 2021;11:2493. 33510235 10.1038/s41598-021-81583-z PMC7843985

[2] Conradt L, Clutton-Brock TH, Thomson D. Habitat segregation in ungulates: are males forced into suboptimal foraging habitats through indirect competition by females? Oecologia. 1999;119:367–77. 28307759 10.1007/s004420050797

[3] Pianka ER, Huey RB. Comparative ecology, resource utilization and niche segregation among gekkonid lizards in the Southern Kalahari. Copeia. 1978;691–701.

[4] Svanbäck R, Bolnick DI. Intraspecific competition drives increased resource use diversity within a natural population. Proc R Soc B Biol Sci. 2007;274:839–44. 10.1098/rspb.2006.0198 PMC2093969 17251094

[5] Urban MC, Tewksbury JJ, Sheldon KS. On a collision course: competition and dispersal differences create no-analogue communities and cause extinctions during climate change. Proc R Soc B Biol Sci. 2012;279:2072–80. 10.1098/rspb.2011.2367 PMC3311897 22217718

[6] Werner EE, Hall DJ, Laughlin DR, Wagner DJ, Wilsmann LA, Funk FC. Habitat partitioning in a freshwater fish community. J Fish Board Can. 1977;34:360–70.

[7] Griffin JN, De La Haye KL, Hawkins SJ, Thompson RC, Jenkins SR. Predator diversity and ecosystem functioning: density modifies the effect of resource partitioning. Ecology. 2008;89:298–305. 18409418 10.1890/07-1220.1

[8] Finke DL, Snyder WE. Niche partitioning increases resource exploitation by diverse communities. Science. 2008;321:1488–90. 18787167 10.1126/science.1160854

[9] Ross ST. Resource partitioning in fish assemblages: a review of field studies. Copeia. 1986;352–88.

[10] Jessopp M, Arneill GE, Nykänen M, Bennison A, Rogan E. Central place foraging drives niche partitioning in seabirds. Oikos. 2020;129:1704–13.

[11] Watabe R, Tsunoda H, Saito MU. Evaluating the Temporal and spatio-temporal niche partitioning between carnivores by different analytical method in Northeastern Japan. Sci Rep. 2022;12:11987. 35835847 10.1038/s41598-022-16020-w PMC9283404

[12] Kronfeld-Schor N, Dayan T. Partitioning of time as an ecological resource. Annu Rev Ecol Evol Syst. 2003;34:153–81.

[13] Macarthur R, Levins R. The limiting similarity, convergence, and divergence of coexisting species. Am Nat. 1967;101:377–85.

[14] Schoener TW. Resource partitioning in ecological communities. Science. 1974;185:27–39. 17779277 10.1126/science.185.4145.27

[15] Reebs SG. Plasticity of diel and circadian activity rhythyms in fishes. Rev Fish Biol Fish. 2002;12:349–71.

[16] Werner EE, Hall DJ. Foraging efficiency and habitat switching in competiting sunfishes. Ecology. 1979;60:256–64.

[17] Van Valen L. Morphological variation and width of ecological niche. Am Nat. 1965;99:377–90.

[18] Bolnick DI, Yang LH, Fordyce JA, Davis JM, Svanbäck R. Measuring individual-level resource specialization. Ecology. 2002;83:2936–41.

[19] Amundsen PA, Bøhn T, Popova OA, Staldvik FJ, Reshetnikov YS, Kashulin NA, et al. Ontogenetic niche shifts and resource partitioning in a Subarctic piscivore fish guild. Hydrobiologia. 2003;497:109–19.

[20] Griffen BD, Byers JE. Partitioning mechanisms of predator interference in different habitats. Oecologia. 2006;146:608–14. 16086166 10.1007/s00442-005-0211-4

[21] Trochine C, Risholt C, Schou MO, Lauridsen TL, Jacobsen L, Skov C et al. Diet and food selection by fish larvae in turbid and clear water shallow temperate lakes. Sci Total Environ. 2022;804. 10.1016/j.scitotenv.2021.150050 34509851

[22] Pekcan-Hekim Z, Hellén N, Härkönen L, Nilsson PA, Nurminen L, Horppila J. Bridge under troubled water: turbulence and niche partitioning in fish foraging. Ecol Evol. 2016;6. 10.1002/ece3.2593 PMC5192875 28035280

[23] Dias RM, Tófoli RM, da Silva JCB, Gomes LC, Agostinho AA. Effects of habitat complexity on trophic interactions of three congeneric fish species. Aquat Ecol. 2022;56:877–89.

[24] Lennox RJ, Westrelin S, Souza AT, Šmejkal M, Říha M, Prchalová M, et al. A role for lakes in revealing the nature of animal movement using high dimensional telemetry systems. Mov Ecol. 2021;9:1–28. 34321114 10.1186/s40462-021-00244-y PMC8320048

[25] Bloomfield EJ, Guzzo MM, Middel TA, Ridgway MS, McMeans BC. Seasonality can affect ecological interactions between fishes of different thermal guilds. Front Ecol Evol. 2022;10:986459.

[26] Copp GH, Robert Britton J, Cucherousset J, García-Berthou E, Kirk R, Peeler E, et al. Voracious invader or benign feline? A review of the environmental biology of European catfish Silurus glanis in its native and introduced ranges. Fish Fish. 2009;10:252–82.

[27] Skov C, Nilsson P. Biology and ecology of Pike. Boca Raton, FL: CRC; 2018.

[28] Vejřík L, Vejříková I, Blabolil P, Eloranta AP, Kočvara L, Peterka J, et al. European catfish (Silurus glanis) as a freshwater apex predator drives ecosystem via its diet adaptability. Sci Rep. 2017;7:15970. 29162872 10.1038/s41598-017-16169-9 PMC5698325

[29] Říha M, Rabaneda-Bueno R, Jarić I, Souza AT, Vejřík L, Draštík V, et al. Seasonal habitat use of three predatory fishes in a freshwater ecosystem. Hydrobiologia. 2022;849:3351–71.

[30] Adámek Z, Mikl L, Šlapanský L, Jurajda P, Halačka K. The diet of predatory fish in drinking water reservoirs – how can they contribute to biomanipulation efforts? Folia Zool. 2019;68:215–24.

[31] Byström P, Karlsson J, Nilsson P, Van Kooten T, Ask J, Olofsson F. Substitution of top predators: effects of pike invasion in a subarctic lake. Freshw Biol. 2007;52:1271–80.

[32] Cucherousset J, Horky P, Slavík O, Ovidio M, Arlinghaus R, Boulêtreau S, et al. Ecology, behaviour and management of the European catfish. Rev Fish Biol Fish. 2018;28:177–90.

[33] Kobler A, Klefoth T, Mehner T, Arlinghaus R. Coexistence of behavioural types in an aquatic top predator: A response to resource limitation? Oecologia. 2009;161:837–47. 19609567 10.1007/s00442-009-1415-9

[34] Říha M, Gjelland K, Děd V, Eloranta AP, Rabaneda-Bueno R, Baktoft H, et al. Contrasting structural complexity differentiate hunting strategy in an ambush apex predator. Sci Rep. 2021;11:17472. 34471177 10.1038/s41598-021-96908-1 PMC8410764

[35] Brodersen J, Howeth JG, Post DM. Emergence of a novel prey life history promotes contemporary sympatric diversification in a top predator. Nat Commun. 2015;6:8115. 26365323 10.1038/ncomms9115

[36] Carol J, Zamora L, García-Berthou E. Preliminary telemetry data on the movement patterns and habitat use of European catfish (Silurus glanis) in a reservoir of the river Ebro, Spain. Ecol Freshw Fish. 2007;16:450–6.

[37] Brevé NWP, Verspui R, de Laak GAJ, Bendall B, Breukelaar AW, Spierts ILY. Explicit site fidelity of European catfish (Silurus Glanis, L., 1758) to man-made habitat in the river meuse, Netherlands. J Appl Ichthyol. 2014;30:472–8.

[38] Vejřík L, Vejříková I, Kočvara L, Sajdlová Z, Hoang The SC, Šmejkal M, et al. Thirty-year-old paradigm about unpalatable perch egg strands disclaimed by the freshwater top-predator, the European catfish (Silurus glanis). PLoS ONE. 2017;12:1–9. 10.1371/journal.pone.0169000 PMC5218473 28060862

[39] Vagnon C, Bazin S, Cattanéo F, Goulon C, Guillard J, Frossard V. The opportunistic trophic behaviour of the European catfish (Silurus glanis) in a recently colonised large peri-alpine lake. Ecol Freshw Fish. 2022;31:650–61.

[40] Encina L, Rodríguez-Ruiz A, Orduna C, Cid JR, de Ilaria M, Granado-Lorencio C. Impact of invasive European catfish (Silurus glanis) on the fish community of Torrejón reservoir (Central Spain) during a 11-year monitoring study. Biol Invasions. 2024;26:745–56.

[41] De Santis V, Brignone S, Čech M, Eckert EM, Fontaneto D, Magalhães F, et al. LIFE PREDATOR: prevent, detect, combat the spread of Silurus glanis in South European lakes to protect biodiversity. NeoBiota. 2024;93:225–44.

[42] Pierce RB. The ecology of Northern pike. Pierce RB, editor. North. Pike Ecol. Conserv. Manag. Hist. University of Minnesota Press; 2012. p. 0.

[43] Říha M, Rabaneda-Bueno R, Lukáš Vejřík|, Jarić, Prchalová M, Šmejkal M et al. Hungry catfish—effect of prey availability on movement dynamics of a top predator. Freshw Biol. 2025;70:e70017.

[44] Vejřík L, Vejříková I, Blabolil P, Sajdlová Z, Kočvara L, Kolařík T, et al. Trophic position of the species and site trophic state affect diet niche and individual specialization: from apex predator to herbivore. Biology (Basel). 2023;12:1113. 37626997 10.3390/biology12081113 PMC10452534

[45] Jarić I, Říha M, Souza AT, Rabaneda-Bueno R, Děd V, Gjelland K, et al. Influence of internal seiche dynamics on vertical movement of fish. Freshw Biol. 2022;67:1543–58.

[46] Ward AJW, Webster MM, Hart PJB. Intraspecific food competition in fishes. Fish Fish. 2006. pp. 231–61.

[47] Winslow L, Read J, Woolway R, Brentrup J, Leach T, Zwart J et al. Package rLakeAnalyzer Lake Phys Tools. 2022.

[48] Wolf N, Carleton SA, Del Martínez C. Ten years of experimental animal isotopic ecology. Funct Ecol. 2009. pp. 17–26.

[49] Boecklen WJ, Yarnes CT, Cook BA, James AC. On the use of stable isotopes in trophic ecology. Annu Rev Ecol Evol Syst. 2011;42:411–40.

[50] Pinnegar JK, Polunin NVC. Differential fractionation of δ13C and δ15N among fish tissues: implications for the study of trophic interactions. Funct Ecol. 1999;13:225–31.

[51] Anderson C, Cabana G. Estimating the trophic position of aquatic consumers in river food webs using stable nitrogen isotopes. J North Am Benthol Soc. 2007;26.

[52] Olsson K, Stenroth P, Nyström P, Granéli W. Invasions and niche width: does niche width of an introduced crayfish differ from a native crayfish? Freshw Biol. 2009;54:1731–40.

[53] Francis RICC. Back-calculation of fish length: a critical review. J Fish Biol. 1990;36:883–902.

[54] Říha M, Ricard D, Vašek M, Prchalová M, Mrkvička T, Jůza T, et al. Patterns in diel habitat use of fish covering the littoral and pelagic zones in a reservoir. Hydrobiologia. 2015;747:111–31.

[55] Moraes K, Souza AT, Vašek M, Bartoň D, Blabolil P, Čech M, et al. Openness of fish habitat matters: lake pelagic fish community starts very close to the shore. Water. 2021;13:3291.

[56] Brooks ME, Kristensen K, van Benthem KJ, Magnusson A, Berg CW, Nielsen A, Skaug HJ, Mächler M, Bolker BM. glmmTMB balances speed and flexibility among packages for zero-inflated generalized linear mixed modeling. R J. 2017;9:378–400.

[57] Lüdecke D. sjPlot: Data visualization for statistics in social science. R Package Version 2018, 2. 2024.

[58] Lenth Remmeans. Estimated Marginal Means, aka Least-Squares Means. R package version 1.10.3. 2024.

[59] Eckrich CA, Albeke SE, Flaherty EA, Bowyer RT, Ben-David M, rKIN. Kernel-based method for estimating isotopic niche size and overlap. J Anim Ecol. 2020;89:757–71. 10.1111/1365-2656.13159 31799690

[60] Swanson HK, Lysy M, POwer M, Stasko AD, Johnson JD, Reist JD. A new probabilistic method for quantifying n-dimensional ecological niches and niche overlap. Ecology. 2015;96:318–24. 26240852 10.1890/14-0235.1

[61] R Development Core Team R. R: a Language and environment for statistical computing. Team RDC, editor. R found. Stat. Comput. R Foundation for Statistical Computing; 2024.

[62] Souchon Y, Tissot L. Synthesis of thermal tolerances of the common freshwater fish species in large Western Europe rivers. Knowl Manag Aquat Ecosyst. 2012;405:03.

[63] Pierce RB, Carlson AJ, Carlson BM, Hudson D, Staples DF. Depths and thermal habitat used by large versus small Northern pike in three Minnesota lakes. Trans Am Fish Soc. 2013;142:1629–39.

[64] Hilge V. The influence of temperature on the growth of the European catfish (Silurus Glanis L). J Appl Ichthyol. 1985;1:27–31.

[65] Nordahl O, Koch-Schmidt P, Tibblin P, Forsman A, Larsson P. Vertical movements of coastal pike (Esox lucius)—On the role of sun basking. Ecol Freshw Fish. 2020;29:18–30.

[66] Skov C, Lucas MC, Jacobsen L. Spatial ecology. In: Skov C, Nilsson PA, editors. Biol Ecol pike. 2018. pp. 91–128.

[67] Říha M, Prchalová M. Models of Animal Distributions in Inland Waters. Encycl Inl Waters, Second Ed. Elsevier; 2022. p. 292–301.

[68] Craig JF. A short review of Pike ecology. Hydrobiologia. 2008;601:5–16.

[69] Beaumont WRC, Hodder KH, Masters JEG, Scott LJ, Welton JS. Activity patterns in pike (Esox lucius), as determined by motion-sensing telemetry. Aquat Telemetry: Adv Appl. Rome: FAO/COISPA, 2005:231–243.

[70] Michels NO, Hrabik TR, Mensinger AF. Effects of predator species, composition and light environment on prey escape behaviours of invasive and native benthic fishes. Ecol Freshw Fish. 2024;33:e12777.

[71] Jepsen N, Beck S, Skov C, Koed A. Behavior of pike (Esox Lucius L.) 50 cm in a turbid reservoir and in a clearwater lake. Ecol Freshw Fish. 2001;10:26–34.

[72] Vejřík L, Vejříková I, Blabolil P, Bartoň D, Sajdlová Z, Kočvara L et al. Long-lines for research monitoring and efficient population regulation of an invasive apex predator, European catfish (Silurus glanis). Heliyon. 2024;10. 10.1016/j.heliyon.2024.e34125 PMC11296022 39100468

[73] Cucherousset J, Olden JD. Ecological impacts of non-native freshwater fishes. Fisheries. 2011;36:215–30.

[74] Eklöv P, Svanbäck R. Predation risk influences adaptive morphological variation in fish populations. Am Nat. 2006;167:440–52. 16673351 10.1086/499544

[75] Huss M, Byström P, Persson L. Resource heterogeneity, diet shifts and intra-cohort competition: effects on size divergence in YOY fish. Oecologia. 2008;158:249–57. 18781331 10.1007/s00442-008-1140-9

[76] Werner EE, Gilliam JF. The ontogenetic niche and species interactions in size-structured populations. Annu Rev Ecol Syst. 1984;15:393–425.

[77] Vašek M, Prchalová M, Peterka J, Ketelaars HAM, Wagenvoort AJ, Čech M, et al. The utility of predatory fish in biomanipulation of deep reservoirs. Ecol Eng. 2013;52:104–11.

[78] Milardi M, Green AJ, Mancini M, Trotti P, Kiljunen M, Torniainen J, et al. Invasive catfish in Northern Italy and their impacts on waterbirds. NeoBiota. 2022;72:109–28.

