# Supplementary material for: Ecosystem, spatial and trophic dimensions of niche partitioning among freshwater fish predators
Source: Mov Ecol. 2025 May 27;13:36. doi: 10.1186/s40462-025-00559-0 (PMC12107910; doi:10.1186/s40462-025-00559-0)
Supplement: Supplementary file 1 — Supplementary Material 1 [file 40462_2025_559_MOESM1_ESM.docx]

# SUPPLEMENTARY MATERIAL

**Ecosystem, spatial and trophic dimensions of niche partitioning among freshwater fish predators**

Milan Říha^+1^, Lukáš Vejřík^1^, Rubén Rabaneda-Bueno^1^, Ivan Jarić^1,2^, Marie Prchalová^1^, Ivana Vejříková ^1^, Marek Šmejkal^1^, Petr Blabolil^1,3^, Martin Čech^1^, Vladislav Draštík^1^, Michaela Holubová^1^, Tomáš Jůza^1^, Karl Ø. Gjelland^4^, Zuzana Sajdlová^1^, Luboš Kočvara^1^, Michal Tušer^1^, Jiří Peterka^1^

# 1. Fish prey availability

Fish prey availability was presented and described in the study of [1]. Short description is given here: Fish prey availability and distribution were assessed using stratified fish community sampling via gillnet. Gillnet sampling, conducted during twilight and night, covered a wide range of available prey across benthic and pelagic habitats. Gillnet surveys were performed in benthic and open water habitats, using 30 m long European standard gillnets with multiple mesh sizes, set overnight [2,3]. Nets were deployed at depths ranging from 0 to >30 m, with separate configurations for benthic (1.5 m high nets) and pelagic (3 m high nets) habitats. Only fish older than YOY were considered in the analysis. Further description in [1].


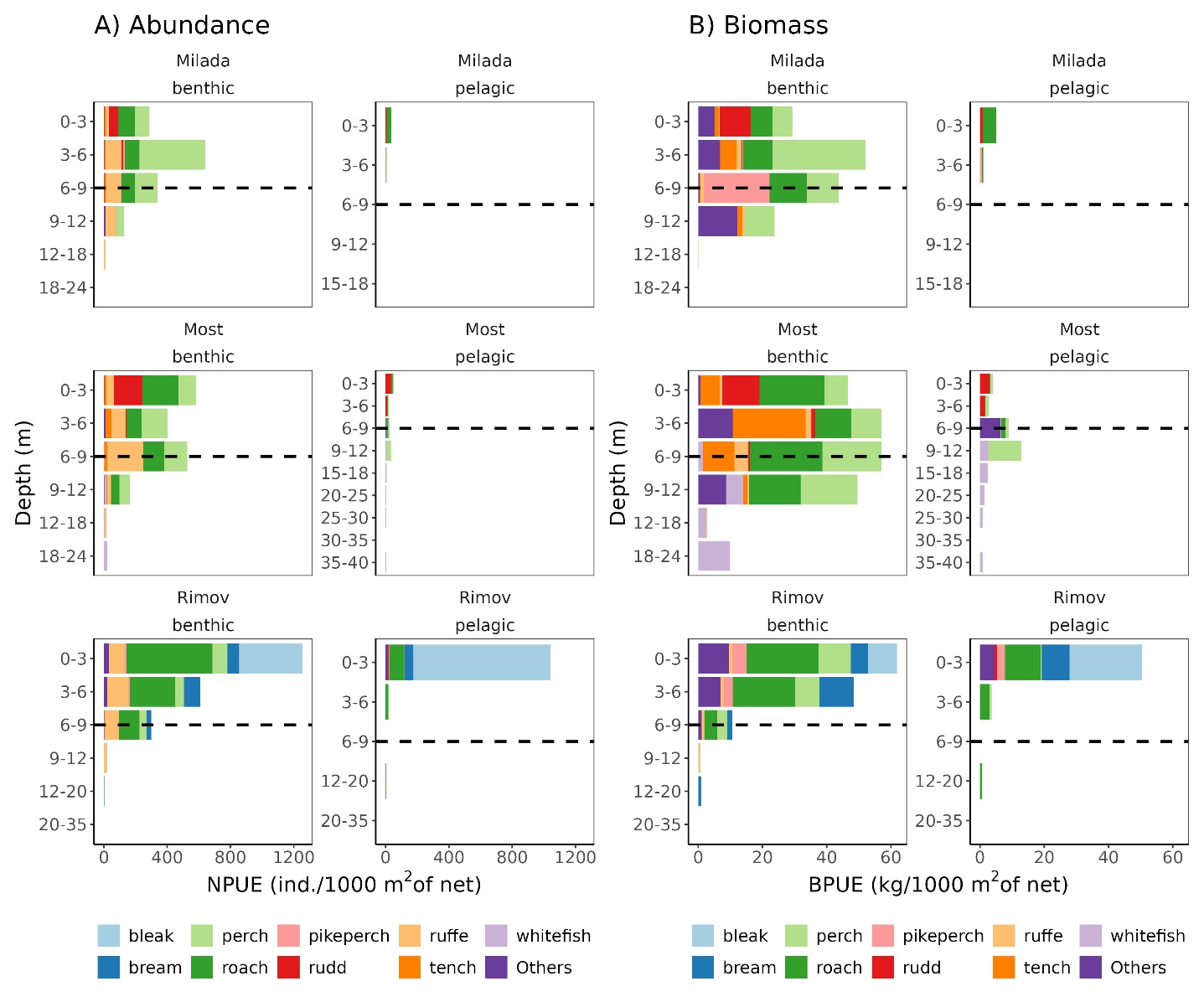


Fig. SM 1. Vertical distribution of fish (A) abundance expressed as NPUE (number of individuals per 1000 m² of net) and (B) biomass expressed as BPUE (kg per 1000 m² of net) in three waterbodies (Milada, Most, and Rimov) in benthic and open water (pelagic) habitats. Bars represent mean values for different fish species across depth strata. The dashed lines indicate the position of the thermocline. Fish species are color-coded according to the legend.

# 2. Telemetry array deployment and telemetry data filtering

The **configuration of the telemetry arrays** and the **shape of the studied waterbodies** are shown in **Figure SM2.**


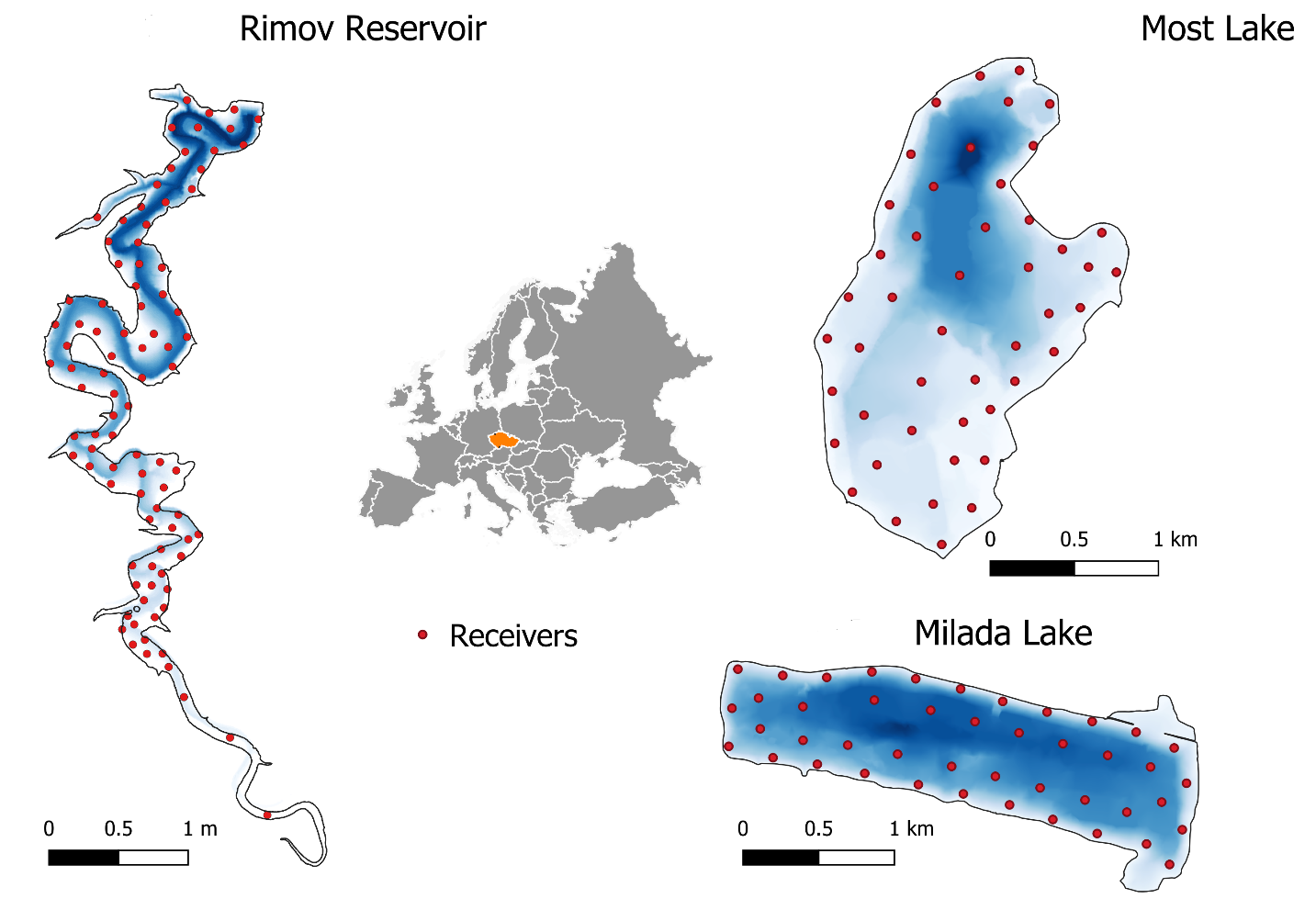


Fig. SM2. Map showing the locations and bathymetry (shaded in blue) of the studied waterbodies, along with the placement of telemetry systems. The Czech Republic, where the research took place, is highlighted in orange. Dots indicate the positions of individual telemetry receivers.

## Filtering of positions estimated by the U-MAP software

U-MAP (User Managed Acoustic Positioning) is a proprietary software from Lotek Inc. that processes transmitter detection data collected with autonomous Lotek WHS-receivers. The detection data recorded by each WHS receiver include tag ID, time of arrival (TOA) and relative signal strength or power of the detected signal. U-MAP uses these data to estimate transmitter positions by means of multilateration on TOA data for transmissions that were detected at three or more receivers. The algorithms solve non-linear equations involving hydrophone positions and the difference in time of signal arrival (TDOA) at each hydrophone detecting the transmission. Depending on the number and geometry of the hydrophones involved in the position calculation, the equation may have multiple solutions ^1^. These are referred to as twin solutions (or shadow solutions). U-MAP saves all twin solutions to the output file, and it is up to the user to choose, or filter, the correct position between the twin positions. Moreover, the use of TDOA implies hyperbolic equations, which also tend to give large errors for certain receiver and transmitter configurations. These configurations depend on the receiver array and the transmitter location, but also on stochastic variation in which receivers that detects the signal. Two successive position estimates may therefore differ in location although the transmitter did not move, if the receivers involved in the position estimate differ. Position estimates produced by programs using TDOA-methods, such as U-MAP, therefore requires filtering by post-processing in order to eliminate erroneous position estimates.

The performance of the position estimates and potential filters were evaluated by short terms tag-tows from boat with high-precision GPS-device above the towed transmitters, by several stationary reference tags and by visual inspections of all fish tracks and filter results.

The filtering procedure required several steps to ensure the best error exclusion:

1. Calculation of detection rate D_r_ and detection mean power D_p_ at each receiver by 20 minute intervals for the transmitter in focus. Detection rate and detection power are expected to be highest closest to the receiver ^2^. These two measures were then combined into one receiver signal scale Ds by scaling each of them before adding them together;
   $D_{s}=scale\left( D_{r} \right)+scale(D_{p})$. Scaling here means centring and dividing by the standard deviation.
2. Calculation of distance weights D_w_ for later use in calculation of regression weights, by the use of the logistic function: $D_{w,i}\frac{1}{{1+2\cdot0.001}^{\frac{(D}{1500}-0.6)}}$, where Di is distance from the receiver i to the position estimate. Parameters where subjectively fitted to obtain a desired logistic shape with high weights to receivers closer than 500 m to the position estimate, and low weight to receivers farther than 1000 m away from the position estimate.
3. Calculation of regression weights R_w_ were then done for each U-MAP position estimate, by first ranking all receivers from highest to lowest Ds value within the current 20 minutes period. The Ds for the highest ranked receiver was then used together with the distance weight Dw for this receiver to obtain _Rw_ through the function $R_{w}=D_{w}(1+D_{s})$
4. Lake shoreline exclusion: All position estimates farther than 50 m outside the lake shoreline polygon were excluded from further filtering, and marked as false position estimate.
5. Calculation of gam-models (generalized additive model^3^) and gam-predictions for east and north-directions. This was done for successive periods of six hours, with one extra hour of data before and after the end of each period included in the gam-regression to stabilize the ends. The resulting gam-models was then used to predict position (Eastgam, Northgam) within the six-hour period at the same time points as the U-MAP estimated positions. Gam residual was calculated as the distance between the U-MAP position estimate and the gam prediction, including residuals for positions excluded from the gam regression. The gam-formulation was y ~ s(time), where y was either east or north, and s was the smooth term specified with a cubic spline regression model. K, the dimension of the basis used to represent the smooth term, was set as a function of the n observations included in the regression period, with K as the nearest integer to n/15, but with a minimum of value of 3.
6. For U-MAP twin positions (i.e. positions with equal timestamp), the position with the lowest gam-residual was flagged as a true position, the other as false.
7. In order to remove heavy outliers with a strong influence on the gam-regression, U-MAP positions with gam-residuals > 1000 m were flagged as false.
8. A second gam-regression was now performed, repeating point 5 but excluding all positions now flagged as false. In addition, positions with gam-residuals > 150 m were excluded from the input data to the second gam regression. New gam-predictions were made for all time points of U-MAP position estimates.
9. For U-MAP twin positions (i.e. positions with equal timestamp), the position with the lowest gam-residual was flagged as a true position, the other as false.
10. Positions with gam-residual larger than 100 m were flagged as false. U-Map position estimates, gam predictions and gam residuals were stored together in the database, such that the gam residual threshold for position acceptance could later be modified if desirable.

# 3. Temperature profile and seasonal development of temperature

Figures SM2 and SM3 provide an overview of the **thermal structure** of the three studied waterbodies (**Milada, Most, and Rimov**) during the study period, highlighting seasonal and temporal temperature variations.

**Figure SM2** illustrates the **seasonal variation in the vertical temperature profile** from June to August. **Figure SM3** presents **temporal changes in water temperature** above and below the thermocline from June to September.


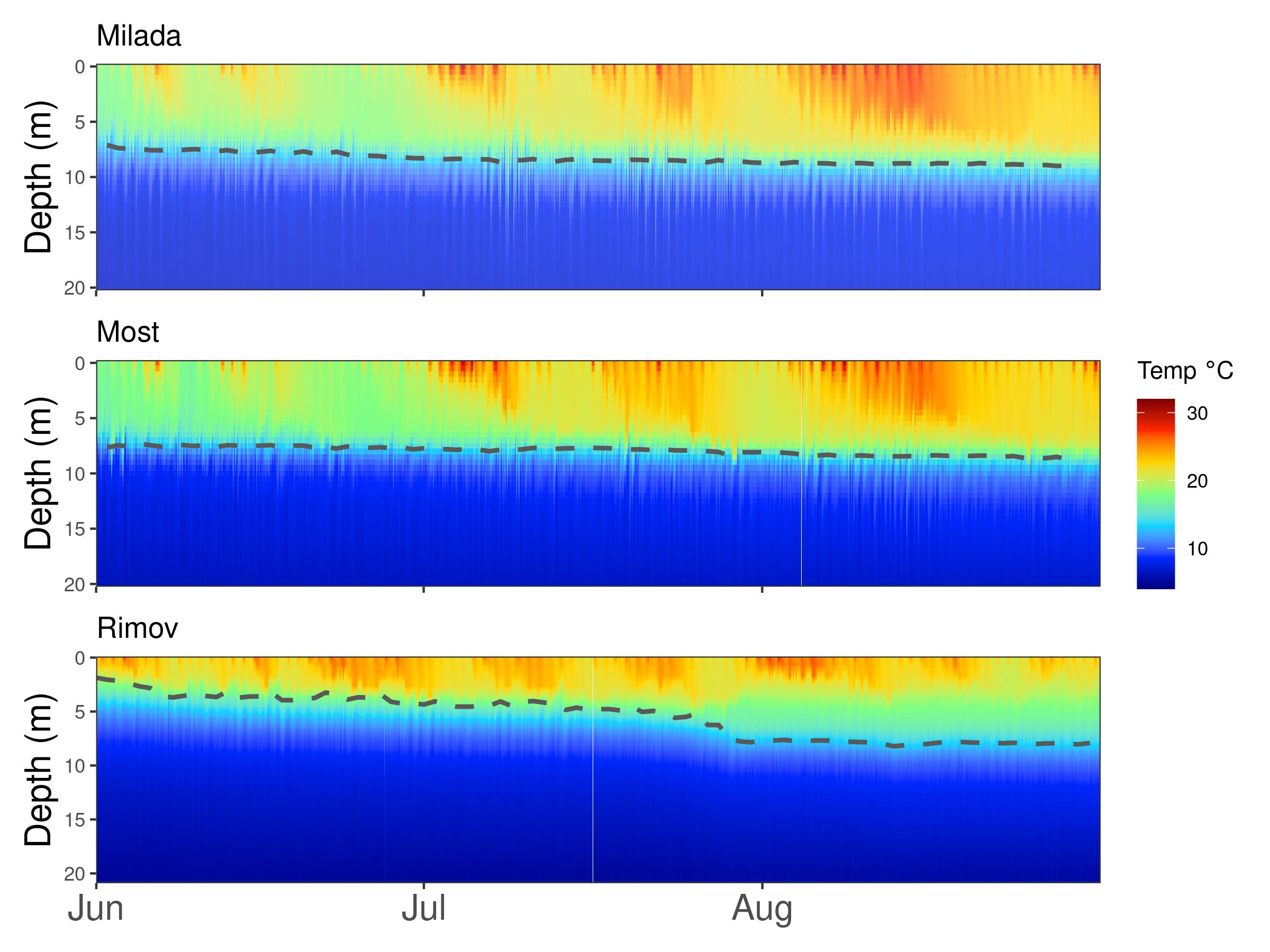


**Figure SM3.** Seasonal variation in the vertical temperature profile (°C) in three waterbodies (Milada, Most, and Rimov) from June to August. The color gradient represents temperature, with blue indicating cooler temperatures and red indicating warmer temperatures. The dashed black line denotes the thermocline.


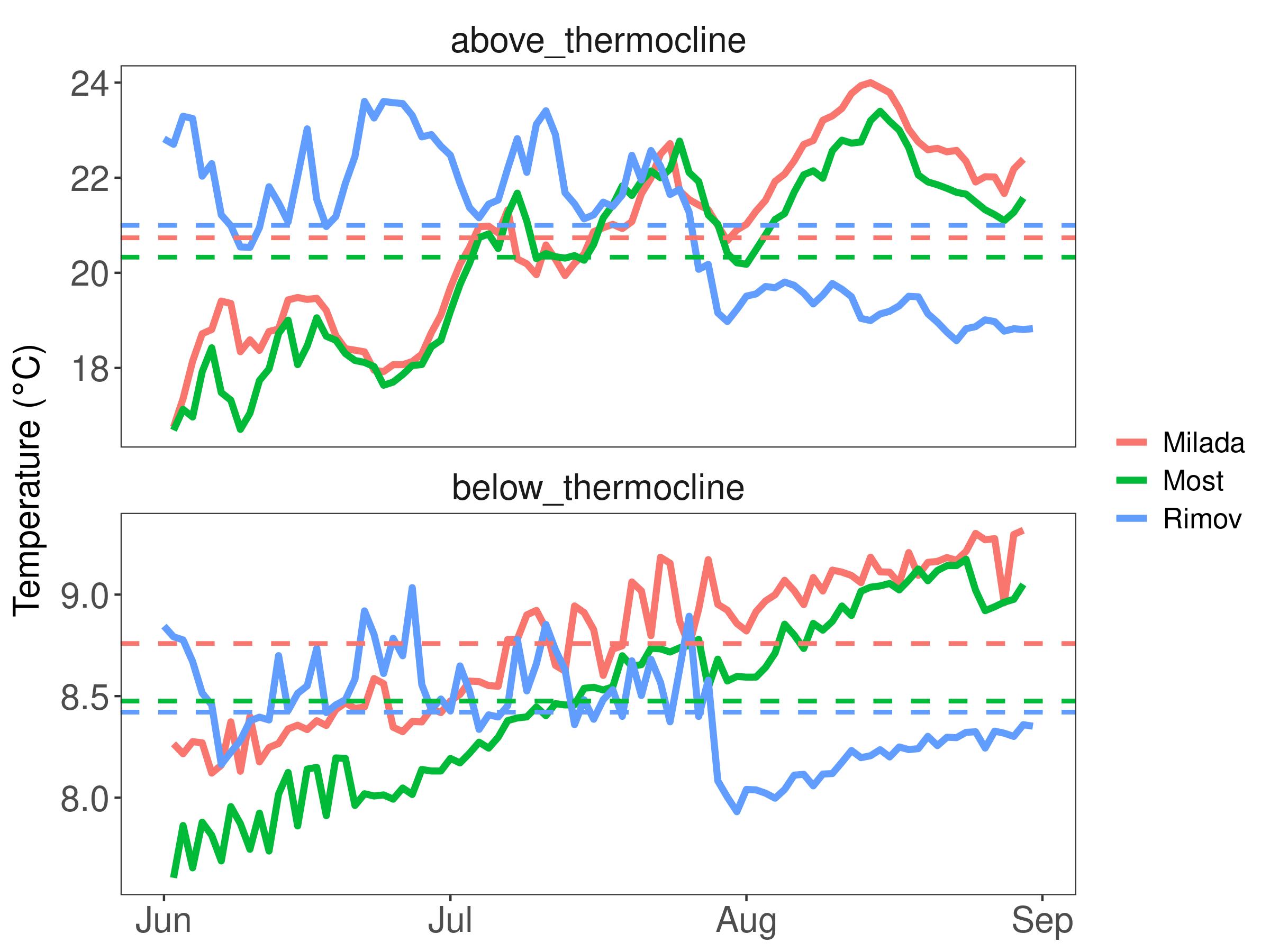


**Figure SM4.** Temporal variation in water temperature (°C) above (top panel) and below (bottom panel) the thermocline in three waterbodies (Milada, Most, and Rimov) from June to September. Solid lines represent daily temperature fluctuations, while dashed lines indicate the mean temperature for each lake.

# 4. Habitat use

The following supplementary figures and table provide detailed statistical results from the **generalized linear mixed-effects model (GLMM)** and associated **contrast analyses.** These analyses explore the effects of **habitat, species, lake, and diel period** on the spatial distribution of European catfish and pike. **Table SM1** presents the full GLMM output, including **main effects and interaction terms** that assess how habitat use is influenced by environmental conditions across lakes and diel periods. To visualize these patterns, **Figures SM5–SM7** show model-predicted **contrasts in habitat use** across different environmental conditions:

- **Figure SM5** illustrates the **estimated differences in habitat use** for catfish and pike across the three waterbodies during **both diel periods.**
- **Figure SM6** depicts **diel differences** in habitat use, comparing catfish (top row) and pike (bottom row) between **day and night** within each lake. This figure highlights habitat shifts and whether species exhibit **significant diel variation.**
- **Figure SM7** presents **pairwise contrasts** of habitat use within each species, separated by **daytime and nighttime** for each lake. This allows for a direct comparison of **within-species habitat use patterns** under different environmental conditions.

**Table SM1.** Results of a **generalized linear mixed-effects model (GLMM)** examining the effects of **habitat, species, lake, and diel period** on habitat use proportions by European catfish and pike. The model includes **all main effects and their interactions,** with **fish ID as a random effect.** Estimates represent **log-odds ratios** with **95% confidence intervals (CI)** and associated **p-values.** Significant effects **(p < 0.05)** are highlighted. **Random effects** include variance components (σ²), intra-class correlation coefficient (ICC), and sample sizes (N). **Marginal R²** indicates variance explained by fixed effects, while **conditional R²** includes both fixed and random effects.

| *Predictor* | *Estimates* | *CI* | *P* | | |
| --- | --- | --- | --- | --- | --- |
| (Intercept) | 2.50 | | 2.01 – 3.12 | **<0.001** | |
| habitat [Ben_Below] | 0.05 | | 0.04 – 0.07 | **<0.001** | |
| habitat [OW_Above] | 0.19 | | 0.15 – 0.23 | **<0.001** | |
| habitat [OW_Below] | 0.05 | | 0.04 – 0.07 | **<0.001** | |
| species [pike] | 1.38 | | 0.99 – 1.92 | 0.057 | |
| lake [Most] | 1.34 | | 0.98 – 1.83 | 0.063 | |
| lake [Rimov] | 0.63 | | 0.45 – 0.88 | **0.006** | |
| diel period [night] | 0.97 | | 0.78 – 1.22 | 0.801 | |
| habitat [Ben_Below] ×species [pike] | 4.06 | | 2.76 – 5.98 | **<0.001** | |
| habitat [OW_Above] ×species [pike] | 0.27 | | 0.19 – 0.38 | **<0.001** | |
| habitat [OW_Below] ×species [pike] | 0.99 | | 0.61 – 1.61 | 0.961 | |
| habitat [Ben_Below] ×lake [Most] | 0.98 | | 0.68 – 1.41 | 0.924 | |
| habitat [OW_Above] × lake[Most] | 0.82 | | 0.59 – 1.14 | 0.239 | |
| habitat [OW_Below] × lake[Most] | 0.92 | | 0.58 – 1.44 | 0.705 | |
| habitat [Ben_Below] ×lake [Rimov] | 3.11 | | 2.10 – 4.60 | **<0.001** | |
| habitat [OW_Above] × lake[Rimov] | 2.78 | | 1.97 – 3.94 | **<0.001** | |
| habitat [OW_Below] × lake[Rimov] | 2.44 | | 1.57 – 3.78 | **<0.001** | |
| species [pike] × lake[Most] | 0.71 | | 0.45 – 1.12 | 0.140 | |
| species [pike] × lake[Rimov] | 0.68 | | 0.42 – 1.10 | 0.118 | |
| habitat [Ben_Below] ×diel period [night] | 0.96 | | 0.64 – 1.46 | 0.856 | |
| habitat [OW_Above] × diel period [night] | 1.14 | | 0.83 – 1.58 | 0.414 | |
| habitat [OW_Below] × diel period [night] | 0.95 | | 0.56 – 1.61 | 0.847 | |
| species [pike] × diel period [night] | 1.01 | | 0.72 – 1.41 | 0.942 | |
| lake [Most] × diel period [night] | 0.67 | | 0.49 – 0.92 | **0.012** | |
| lake [Rimov] × diel period [night] | 0.62 | | 0.44 – 0.86 | **0.005** | |
| (habitat [Ben_Below] × species [pike]) × lake [Most] | 0.51 | | 0.30 – 0.87 | **0.014** | |
| (habitat [OW_Above] ×species [pike]) × lake[Most] | 2.96 | | 1.81 – 4.84 | **<0.001** | |
| (habitat [OW_Below] ×species [pike]) × lake[Most] | 1.92 | | 0.98 – 3.78 | 0.058 | |
| (habitat [Ben_Below] ×species [pike]) × lake[Rimov] | 0.29 | | 0.17 – 0.52 | **<0.001** | |
| (habitat [OW_Above] ×species [pike]) × lake[Rimov] | 1.82 | | 1.09 – 3.04 | **0.022** | |
| (habitat [OW_Below] ×species [pike]) × lake[Rimov] | 1.72 | | 0.89 – 3.35 | 0.108 | |
| (habitat [Ben_Below] ×species [pike]) × diel period [night] | 1.79 | | 1.00 – 3.21 | **0.049** | |
| (habitat [OW_Above] ×species [pike]) × diel period [night] | 0.97 | | 0.60 – 1.58 | 0.904 | |
| (habitat [OW_Below] ×species [pike]) × diel period [night] | 1.11 | | 0.50 – 2.44 | 0.805 | |
| (habitat [Ben_Below] ×lake [Most]) × diel period [night] | 1.39 | | 0.79 – 2.47 | 0.254 | |
| (habitat [OW_Above] ×lake [Most]) × diel period [night] | 1.91 | | 1.20 – 3.02 | **0.006** | |
| (habitat [OW_Below] ×lake [Most]) × diel period [night] | 1.43 | | 0.67 – 3.03 | 0.353 | |
| (habitat [Ben_Below] × lake [Rimov]) × diel period [night] | 1.42 | | 0.80 – 2.53 | 0.229 | |
| (habitat [OW_Above] ×lake [Rimov]) × diel period [night] | 2.01 | | 1.24 – 3.27 | **0.005** | |
| (habitat [OW_Below] ×lake [Rimov]) × diel period [night] | 1.53 | | 0.78 – 3.02 | 0.220 | |
| (species [pike] × lake[Most]) × diel period[night] | 1.20 | | 0.76 – 1.91 | 0.432 | |
| (species [pike] × lake[Rimov]) × diel period[night] | 2.32 | | 1.40 – 3.85 | **0.001** | |
| (habitat [Ben_Below] ×species [pike] × lake[Most]) × diel period[night] | 0.67 | | 0.30 – 1.48 | 0.320 | |
| (habitat [OW_Above] ×species [pike] × lake[Most]) × diel period[night] | 0.48 | | 0.24 – 0.95 | **0.035** | |
| (habitat [OW_Below] ×species [pike] × lake[Most]) × diel period[night] | 0.79 | | 0.28 – 2.27 | 0.667 | |
| (habitat [Ben_Below] ×species [pike] × lake[Rimov]) × diel period[night] | 0.32 | | 0.14 – 0.73 | **0.007** | |
| (habitat [OW_Above] ×species [pike] × lake[Rimov]) × diel periodnight] | 0.27 | | 0.13 – 0.55 | **<0.001** | |
| (habitat [OW_Below] ×species [pike] × lake[Rimov]) × diel period[night] | 0.30 | | 0.11 – 0.83 | **0.021** | |
| **Random Effects** | | | | |  |
| σ^2^ | 0.44 | | | |  |
| τ_00_ _fishid_ | 0.09 | | | |  |
| ICC | 0.17 | | | |  |
| N _fishid_ | 84 | | | |  |
| Observations | 6053 | | | |  |
| Marginal R^2^ / Conditional R^2^ | 0.682 / 0.736 | | | |  |


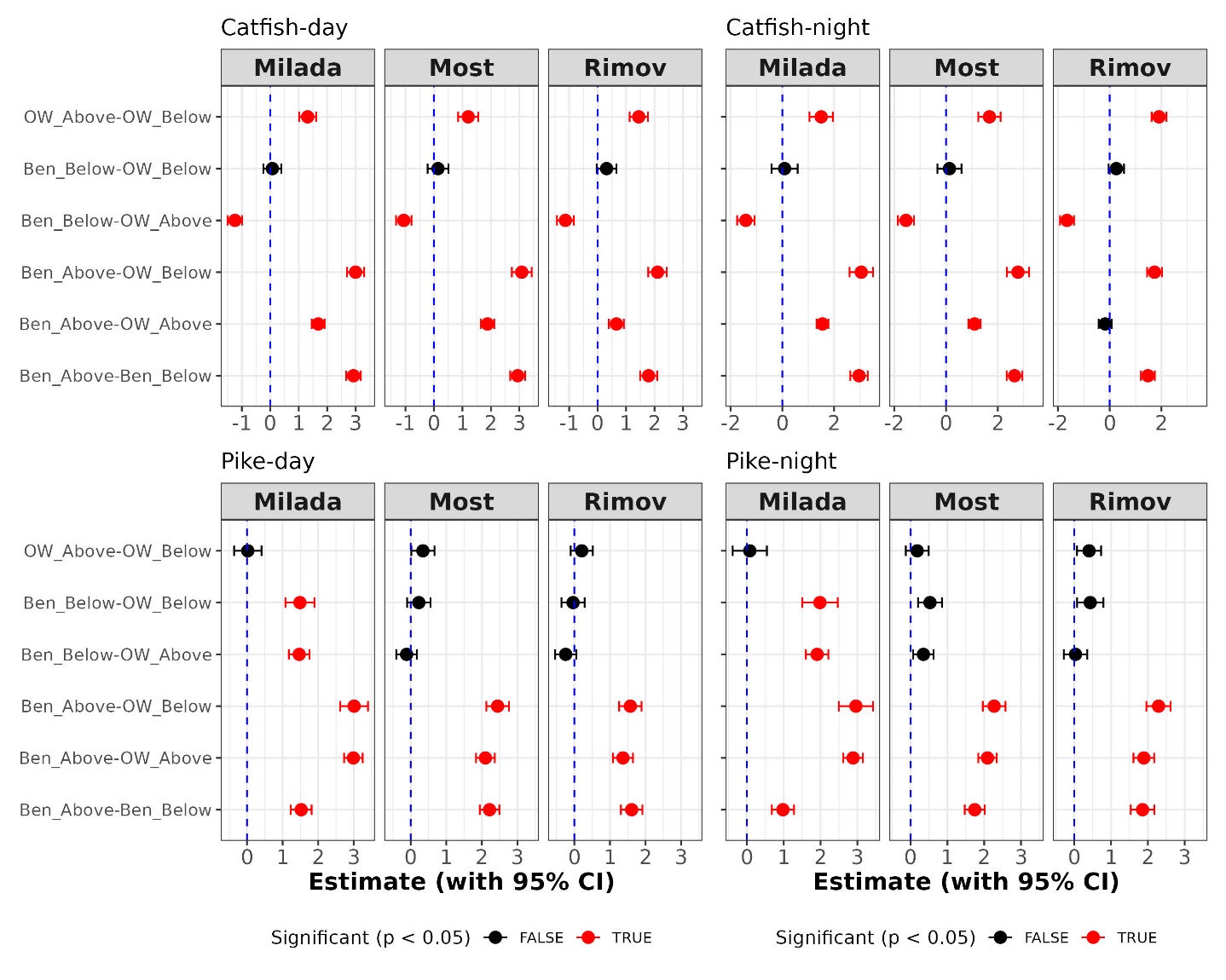


**Figure SM5.** Pairwise contrasts of habitat use within species (European catfish and pike) during daytime and nighttime separately for each waterbody (Milada, Most, and Rimov). The x-axis represents the estimated effect size with 95% confidence intervals (CI), while the y-axis denotes habitat contrasts. The top row shows **catfish habitat contrasts** for daytime (left) and nighttime (right), while the bottom row presents **pike habitat contrasts** for the same periods. Red points indicate **significant differences** (p < 0.05), while black points represent **non-significant results.** The vertical **blue dashed line at x = 0** represents the null effect line, where no difference between habitats is expected.


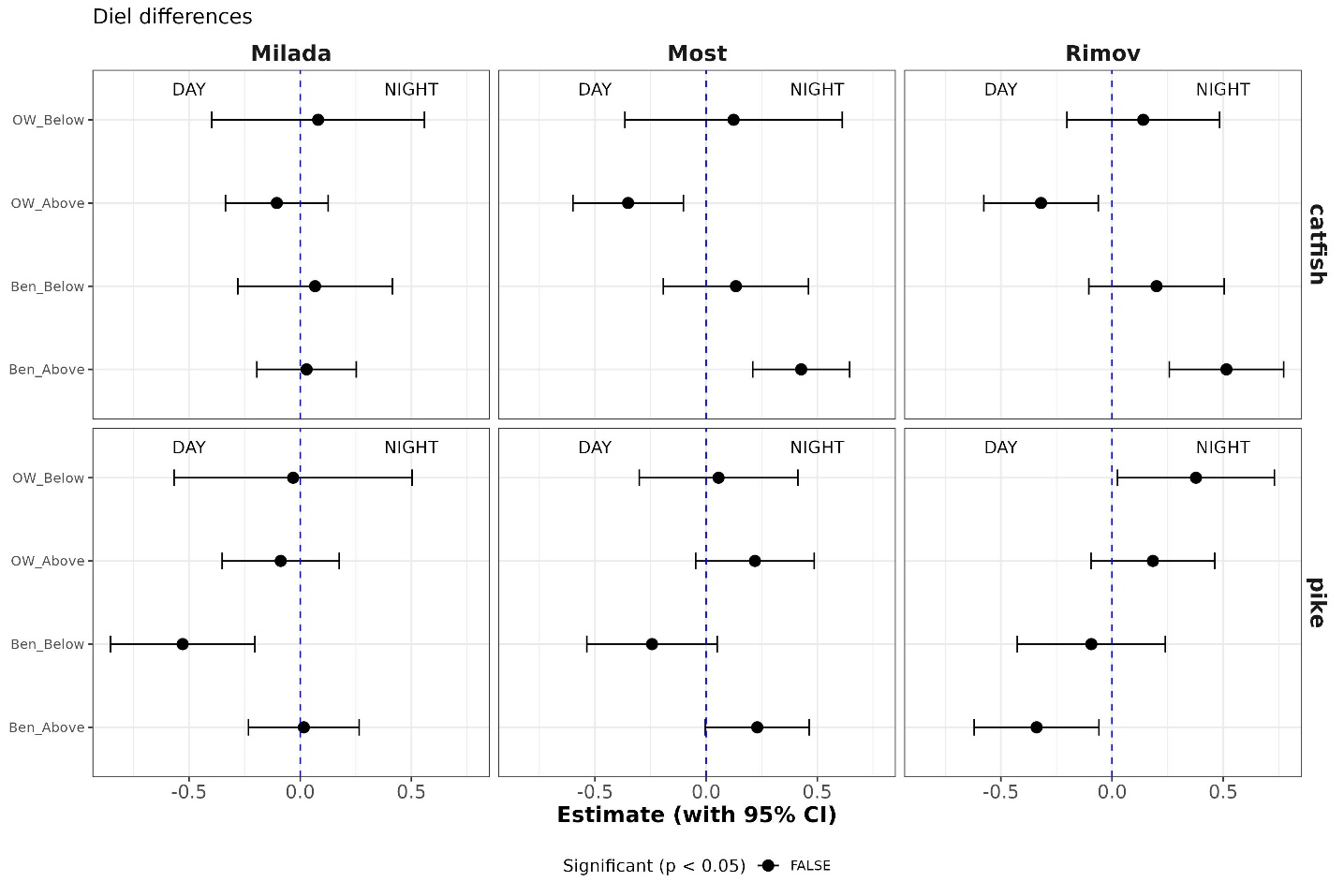


**Figure SM6.** Diel differences in habitat use for pike and European catfish across three waterbodies (Milada, Most, and Rimov). The x-axis represents the estimated effect size with 95% confidence intervals (CI). The y-axis denotes different habitat types: **Ben_Above** (benthic above), **Ben_Below** (benthic below), **OW_Above** (open water above), and **OW_Below (**open water below). The upper row represents catfish, while the lower row represents pike. Each panel compares habitat use between **day and night,** with labels positioned above the lowest habitat category. The vertical **blue dashed line at x = 0** represents the null effect line, indicating no diel difference. Black points represent **non-significant** estimates (p ≥ 0.05).


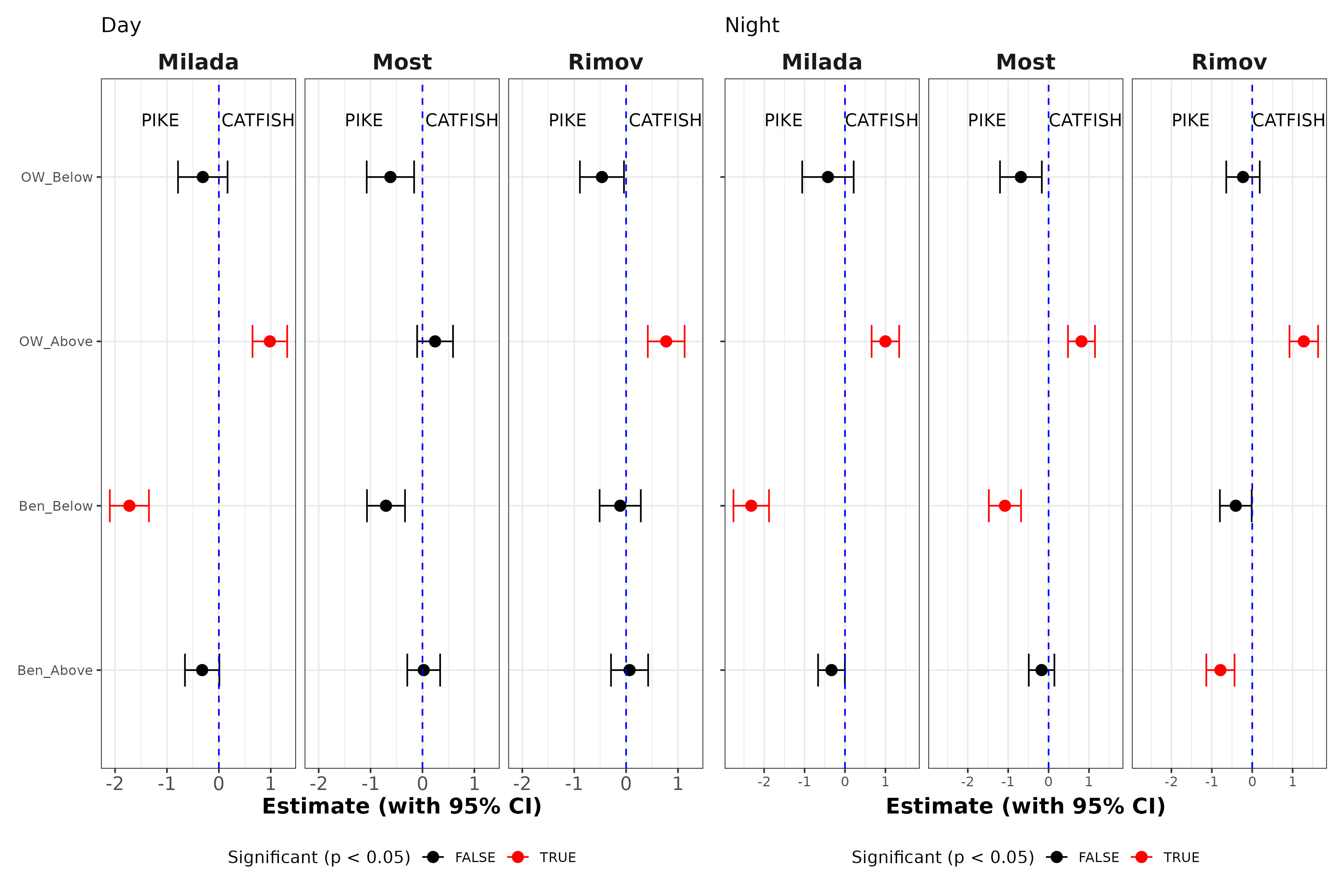


**Figure SM7.** Estimated differences in habitat use for pike and European catfish across three waterbodies (Milada, Most, and Rimov) during nighttime. The x-axis represents the estimated effect size with 95% confidence intervals (CI). Different habitat types are shown on the y-axis: **Ben_Above** (benthic above), **Ben_Below** (benthic below), **OW_Above** (open water above), and **OW_Below** (open water below). Black points represent non-significant estimates (p ≥ 0.05), while red points indicate significant differences (p < 0.05). The vertical blue dashed line at **x = 0** represents the null effect line. Labels "PIKE" and "CATFISH" denote species comparisons in each lake.

References

1. Říha M, Rabaneda-Bueno R, Lukáš Vejřík |, Jarić | Ivan, Prchalová M, Šmejkal M, et al. Hungry catfish—effect of prey availability on movement dynamics of a top predator. Freshw Biol. 2025;70:e70017.

2. Říha M, Gjelland K, Děd V, Eloranta AP, Rabaneda-Bueno R, Baktoft H, et al. Contrasting structural complexity differentiate hunting strategy in an ambush apex predator. Sci Rep. 2021;11:17472.

3. Tesfaye GC, Souza AT, Bartoň D, Blabolil P, Čech M, Draštík V, et al. Long-term monitoring of fish in a freshwater reservoir: Different ways of weighting complex spatial samples. Front Environ Sci. 2022;10:1000087.
